# Maternal knowledge of obstetric danger signs and related factors among antenatal care attendees in Hargeisa, Somaliland: A cross-sectional analysis

**DOI:** 10.1371/journal.pone.0353778

**Published:** 2026-07-15

**Authors:** Abdeta Muktar Ahmed, Mohamed Abdilahi Ahmed, Abdulkadir Mohammed Nuh, Hamse Khalif Hassan

**Affiliations:** 1 Addis Ababa Medical University College, Hargeisa Campus, Department of Public Health, Hargeisa, Somaliland; 2 Addis Ababa Medical University College, Hargeisa Campus, Department of Nutrition, Hargeisa, Somaliland; 3 International Health program, College of Medicine, National Yang Ming Chiao Tung University (NYCU), Taipei, Taiwan; Federal Medical Centre Birnin Kudu, NIGERIA

## Abstract

**Background:**

Maternal mortality remains a major global health challenge, disproportionately affecting low- and middle-income countries. Despite international efforts, nearly 95% of maternal deaths occur in these regions, with Sub-Saharan Africa bearing the highest burden. Limited knowledge of obstetric danger signs is a key factor contributing to delays in seeking care, which in turn increases the risk of preventable maternal complications. Evidence on maternal awareness in Somaliland is scarce.

**Objective:**

This study aimed to assess the level of knowledge of obstetric danger signs and associated factors among pregnant women attending antenatal care in Hargeisa, Somaliland.

**Methods:**

A facility-based cross-sectional study was conducted from July 2 to August 4, 2022, among pregnant women attending antenatal care services. A total of 222 participants were selected using a systematic random sampling technique from eight maternal and child health centers. Data were collected through face-to-face interviews using a pretested and structured questionnaire and analyzed with SPSS version 25. Bivariate and multivariate logistic regression was carried out.

**Results:**

About 35.6% had a good knowledge specifically about pregnancy related obstetric danger signs. Only 57 women (25.7%) demonstrated overall good knowledge of obstetric danger signs across pregnancy, childbirth, and the postpartum periods. Knowledge was highest for vaginal bleeding (65% − 67%), while less visible complications such as convulsions and blurred vision were less frequently mentioned. Multivariate analysis revealed that secondary level education or higher (AOR = 3.6, 95% CI: 1.8–7.3), access to and usage of media (AOR = 2.6, 95% CI: 1.2–5.6), and institutional delivery (AOR = 3.4, 95% CI: 1.6–7.2), were significant predictors of knowledge.

**Conclusion:**

Maternal knowledge of obstetric danger signs in Hargeisa was low. Targeted health education, broader use of mass media, and promoting institutional delivery are critical strategies to improve and reduce preventable maternal mortality.

## Background

Maternal mortality remains a pressing global issue. In 2023, more than 700 women lost their lives to pregnancy-related and childbirth-related avoidable causes every day, or roughly one woman every two minutes [1]. Despite international efforts to address it, maternal mortality is still a serious global health concern. The startling truth is that the majority of maternal fatalities are avoidable and result from severe health disparities that occur both within and between nations and worldwide. These disparities are significant. Nearly 95% of all maternal deaths take place in low- and middle-income nations, with Sub-Saharan Africa bearing the highest risk, accounting for 70% of all maternal deaths. The 2030 United Nations Sustainable Development Goal (SDG), which aims to lower the rate of maternal fatalities to fewer than 70 per 100,000 live births, is still significantly above the current global rate of maternal deaths (223 per 100,000 live births) [2–3].

Although an estimated 260,000 maternal deaths, or about 700 deaths per day, occurred globally in 2023, maternal mortality has decreased by 40% since 2000, which is indicative of ongoing international efforts to provide access to basic healthcare services. No region was designated as having a very high maternal mortality ratio (MMR), and no country was predicted to have an exceptionally high MMR for the first time [1].

One of the main causes of maternal mortality in the underdeveloped world is lack of awareness among women, families, and birth attendants about the obstetric warning signs [4]. This challenge can be better understood within the framework of the Three Delays Model, which highlights delays in (a) deciding to seek care, (b) reaching a health facility, and (c) receiving appropriate care upon arrival [5]. In this context, inadequate knowledge of obstetric danger signs primarily contributes to the first delay, as women and their families may fail to recognize life-threatening complications and seek timely medical attention. These deaths can be prevented through educating women about the warning signs of obstetric dangers, encouraging them to seek medical attention when necessary, and counselling them on these obstetric danger signs, as well as the right management of the complications [4].

Obstetric danger signs are warning indications that could point to potentially life-threatening conditions during pregnancy, labor, or the postpartum phases. Approximately 75% of maternal deaths during these three phases are caused by obstetric danger signs, which include vaginal bleeding, blurred vision, lack of fetal movement, a gush of fluid from the vagina, high fever, retained placenta, foul-smelling discharge, convulsions, and severe headaches. Because mothers and family members may quickly recognize these signs without need for a complex professional diagnosis, raising maternal knowledge is a crucial approach in minimizing care-seeking delays [6]. Pregnant women who notice warning symptoms and seek prompt obstetric care could reduce maternal mortality from complications connected to childbirth in low-income countries. One of the most frequent reasons why people fail to detect complications when they arise and put off seeking care is a lack of warning signs [7]. Poor maternal and newborn outcomes result from pregnant women’s lack of awareness of obstetric danger signs, which is a major cause in delays in seeking care. Designing focused treatments that raise awareness, encourage prompt care-seeking behavior, and improve birth preparedness requires an understanding of the factors influencing pregnant women’s knowledge of obstetric danger signs [8].

According to the 2020 Somaliland Health and Demographic Survey (SLHDS), the country experiences an estimated 396 maternal deaths for every 100,000 live births. Furthermore, only 47% of pregnant women reported attending at least one antenatal care (ANC) visit, and merely 33% delivered with the assistance of skilled birth attendant [9]. Although Somaliland’s maternal mortality ratio has shown a declining trend and is not among the highest in the region, it still remains far from achieving the SDG target of reducing maternal mortality to 70 per 100,000 live births by 2030.

To date, no studies have been conducted in Hargeisa, Somaliland, to assess the level of knowledge regarding obstetric danger signs and their associated factors. Understanding this knowledge gap is crucial, as improving awareness of obstetric danger signs can inform strategies to enhance maternal and health outcomes and reduce maternal mortality. Therefore, the present study aimed to evaluate the level of knowledge of obstetric danger signs and to identify the associated factors among pregnant women attending antenatal care (ANC) services in selected health institutions in Hargeisa, Somaliland.

## Materials and methods

### Study design and area

A facility-based cross-sectional study was conducted in Hargeisa, the capital of Somaliland, from July 2 to August 4, 2022. Somaliland, a self-declared autonomous state in the Horn of Africa that unilaterally separated from Somalia in 1991, had an estimated population of 6.2 million [10]. Hargeisa, its capital, is home to approximately 1.8 million people [11].

### Study population

The source population consisted of pregnant women attending ANC services at health institutions in Hargeisa during the study period.

**Inclusion criteria**: All pregnant women attending antenatal care services in the selected health institutions during the study period who were physically and mentally able and willing to participate.

**Exclusion criteria**: Pregnant women who were critically ill at the time of data collection, defined as those experiencing severe medical or obstetric conditions that impaired their ability to communicate or safely participate in an interview.

The sample size was calculated using a single population proportion formula:


$$\begin{array}{c} n\;=\;(Z^{\text{2}}\;x\;p\;x\;(\text{1}-p))\;/\;d^{\text{2}} \end{array}$$

Where Z = 1.96 (95% confidence level), p = 0.155 (15.5% prevalence of knowledge of obstetric danger signs from a previous study [12]), and 5% margin of error.


$$\begin{array}{c} \text{n }=\;({\text{1}.\text{9}\text{6}}^{\text{2}}\;x\;\text{0}.\text{1}\text{5}\text{5}\;x\;\text{0}.\text{8}\text{4}\text{5})\;/\;({\text{0}.\text{0}\text{5}}^{\text{2}})\;\approx \;\text{2}\text{0}\text{1} \end{array}$$

After adding a 10% non-response rate, the final sample size became 222.

### Sampling procedure

Eight centers were selected through simple random sampling from the total of 17 Maternal and Child Health (MCH) centers to ensure representativeness and minimize selection bias.

The selected health facilities were public Maternal and Child Health (MCH) centers that provide comprehensive primary healthcare services, including antenatal care, delivery services, postnatal care, and child health services. In addition, routine maternal services including health education, nutritional counselling, basic laboratory investigations, and clinical examinations are offered. The facilities are staffed by trained healthcare professionals, including midwives and nurses. As government-run institutions, they follow standardized service delivery protocols, ensuring relatively uniform care across facilities. These MCH centers are widely distributed throughout Hargeisa city, making them highly accessible and commonly utilized by the population.

Proportional allocation was applied to distribute the study units across the selected facilities, based on the number of ANC attendees recorded in the month preceding the survey. The sampling interval (K) was calculated as about three, meaning every third eligible woman attending ANC was invited to participate until the allocated quota for each facility was reached.

### Research instrument and measurement

Data were collected using pretested, structured, interviewer-administered questionnaire adapted and modified from the Maternal and Newborn Health Program of JHPIEGO, an affiliate of Johns Hopkins University, and other related articles [13–14]. The tool comprised four sections: (1) Sociodemographic characteristics, (2) Reproductive and obstetric history, (3) Service utilization, and (4) Knowledge of obstetric danger signs. The questionnaire was initially prepared in English, translated into Somali, and subsequently back-translated into English to ensure consistency and accuracy.

Women’s knowledge of obstetric danger signs was assessed by asking participants to spontaneously mention danger signs that may occur during pregnancy, childbirth, and the postpartum period, which were categorized into three groups. Knowledge of pregnancy danger signs (the study’s dependent variable) was defined as correctly identifying at least three key signs, including vaginal bleeding, swollen hands/face, and blurred vision. Knowledge of childbirth/labor danger signs was defined as mentioning at least three major signs, namely vaginal bleeding, prolonged labor, convulsions, or retained placenta. For the postpartum period, women were regarded as having good knowledge if they identified at least three major signs, including vaginal bleeding after delivery, loss of consciousness, and postpartum fever. Overall, a woman was considered as having good knowledge on obstetric danger signs if she was able to mention at least three key signs across each of the three phases, without prompting, in line with the JHPIEGO maternal health framework [13,14]. Those who mentioned fewer than three key danger signs were categorized as having poor knowledge. Hence, for analysis, the dependent variables were dichotomized into “**Good knowledge**” and “**Poor knowledge**.” This classification approach has been widely used in similar studies assessing maternal knowledge of obstetric danger signs in low- and middle-income settings.

### Data collection procedures

Data were collected through face-to-face interviews conducted by six trained BSc Nursing students. Two experienced supervisors oversaw the process. Prior to data collection, the questionnaire was pretested on 22 pregnant women at an MCH center outside the study area. Based on pretest findings, adjustments were made to improve clarity and accuracy. Training was provided to data collectors and supervisors on the objectives of the study, the data collection tool, ethical considerations, and procedures to ensure confidentiality.

### Data processing and analysis

Completed questionnaires were checked daily for completeness and consistency. Data were cleaned, coded, and entered into SPSS version 25 for analysis. Descriptive statistics were used to summarize the data. Bivariate logistic regression was first performed, and variables with a p-value < 0.2 were considered candidates for multivariate analysis. Subsequently, multivariate logistic regression was carried out to examine associations between independent variables and the outcome variable while controlling for potential confounding factors. Adjusted odds ratios (AOR) with 95% confidence interval (CI) were computed to determine the strength of associations. Statistical significance was declared at p < 0.05.

### Ethical considerations

Ethical approval for the study was obtained from the Research Ethics Committee of Addis Ababa Medical University College, Hargeisa campus (Ref. No: AAMUCHC/REC/87/22). The purpose of the study was explained to all participants, and written informed consent was obtained prior to data collection. Participation was voluntary, and respondents were assured that their information would be used solely for research purpose and kept confidential. Names and personal identifiers were not recorded to preserve anonymity.

## Result

### Socio-demographic characteristics

A total of 222 pregnant women participated in the study, yielding a response rate of 100%. The mean age of the respondents was 28.3 years (+6.5 SD), with a median age of 28.5 years, ranging from 17 to 43 years. More than half of the respondents (55.4%) were between 25 and 36 years of age. Most participants 201 (90.5%) were currently married. Regarding educational attainment, 75 (33.8%) had no formal education, 53 (23.9%) had completed primary education, 38 (17.1%) had secondary education, and 56 (25.2%) attained higher education. By contrast, more than half of their spouses 121 (54.5%) had higher education, while only 59 (26.6%) had primary or no formal education. In terms of occupation, the majority of respondents 153 (68.9%) were merchants, 49 (22.1%) were housewives/unemployed, and 20 (9%) were formally employed. Among spouses, 149 (67.1%) were employed, 62 (27.9%) were merchants, and 11 (5%) were unemployed. The mean monthly household income was 561 USD. A large proportion 152 (68.5%) reported monthly incomes of <500 USD. Additionally, 142 respondents (64%) had access to mainstream or social media (Table 1).

**Table 1:** Socio-demographic characteristics of the respondents (n = 222).

Variables	Category	Frequency (n)	Percentage (%)
Age (years)	≤24	70	31.5
	25-36	123	55.4
	≥37	29	13.1
Marital status	Currently married	201	90.5
	Widowed/Divorced	21	9.5
Educational status (women)	No formal education	75	33.8
	Primary	53	23.9
	Secondary	38	17.1
	Higher education	56	25.2
Educational status (spouse)	No formal education	36	16.2
	Primary	23	10.4
	Secondary	42	18.9
	Higher education	121	54.5
Occupation (women)	Merchant	153	68.9
	Housewife/Unemployed	49	22.1
	Employed	20	9
Occupation (Spouse)	Employed	149	67.1
	Merchant	62	27.9
	Unemployed	11	5
Monthly family income (USD)	≤500	152	68.5
	501-1000	30	13.5
	>1000	40	18
Access to media	Yes	142	64
	No	80	36

### Obstetric characteristics

The age at first marriage ranged from 14 to 28 years, with a mean of 18.9 years. Half of the respondents 111 (50%) married between the age of 14–18 years. The age at first pregnancy ranged from 16 to 30 years, with a mean of 20 years; more than half 119 (53.6%) conceived between 19–24 years. At the time of data collection, nearly half of the respondents 102 (45.9%) were in their third trimester.

Number of pregnancies ranged from 1 to 11, with a mean of 4.5, of which 95 (42.8%) had experienced 4–6 pregnancies. Meanwhile, 136 participants (61.3%) reported having up to three births. Regarding pregnancy intention, 144 (64.9%) stated their current pregnancy was planned, while 78 (35.1%) described otherwise. The majority of participants, 178 (80.2%) reported not experiencing any obstetric danger signs during their current pregnancy (Table 2).

**Table 2:** Obstetric variables of the study participants (n = 222).

Variables	Category	Frequency (n)	Percent (%)
Age at first marriage (years)	14-18	111	50
	19-23	92	41.4
	≥24	19	8.6
Age at first pregnancy (years)	16-19	108	48.6
	20-23	84	37.8
	≥24	30	13.5
Trimester (Current pregnancy)	First	54	24.5
	Second	66	29.7
	Third	102	45.9
Number of pregnancies	≤3	83	37.4
	4-6	95	42.8
	>6	44	19.8
Number of births	≤3	136	61.3
	4-6	62	27.9
	>6	24	10.8
Current pregnancy intended	Yes	144	64.9
	No	78	35.1
History of danger signs	Yes	44	19.8
	No	178	80.2

### Maternal health service utilization

With respect to ANC visits during the current pregnancy, 63 women (28.4%) had attended only once, while 66 (29.7%), 55 (24.8%), and 38 (17.2%) had attended two, three, and four or more visits, respectively. More than two-thirds 158 (71.2%) had received health education about obstetric danger signs during ANC visits, whereas 64 (28.8%) had not.

Among 209 respondents with previous childbirth experience, 128 (61.2%) delivered their last baby in a health institution, while 81 (38.8%) delivered at home. The majority 187 (89.5%) reported spontaneous vaginal delivery, followed by 15 (7.2%) instrumental delivery, and 7 (3.3%) cesarean section.

The reported travel time to the nearest health facility ranged from 1 to 120 minutes, with a mean of 33 minutes. More than half, 130 (58.6%) lived within a 30-minutes walking distance. Regarding decision-making autonomy, 116 (52.3%) reported making decisions independently, 82 (36.9%) jointly with their partner, and 24 (10.8%) reported that decisions were solely by their partner. Only 85 (38.3%) discussed maternal health service utilization with their spouses, while the majority, 137 (61.7%) did not (Table 3).

**Table 3:** Maternal health service utilization (n = 222).

Variables	Category	Frequency (n)	Percentage (%)
ANC visits (current pregnancy)	1-3	184	82.9
	≥4	38	17.2
Received health education on danger signs	Yes	158	71.2
	No	64	28.8
Place of last delivery (n = 209)	Home	81	38.8
	Health institution	128	61.2
Mode of delivery (n = 209)	Spontaneous vaginal delivery (SVD)	187	89.5
	Instrumental Delivery	15	7.2
	Cesarean section	7	3.3
Time to nearest health facility (walk)	≤30 min	130	58.6
	>30 min	92	41.4
Decision making power	Self	116	52.3
	Joint (both)	82	36.9
	Partner only	24	10.8
Discussion with spouse on maternal services	Yes	85	38.3
	No	135	61.7

### Knowledge of obstetric danger signs

Respondents were asked to list key obstetric danger signs spontaneously. During pregnancy, the most commonly mentioned signs were vaginal bleeding by 144 participants (64.9%), blurred vision 126 (56.8%), swollen face and limbs 119 (53.6%), severe headache 108 (48.6%), hypertension 75 (33.8%), and convulsion 73 (32.9%).

During childbirth, prolonged labor by 150 respondents (67.6%) and vaginal bleeding by 149 (67.1%) were the most frequently mentioned danger signs, followed by retained placenta 115 (51.8%), hypertension 96 (43.2%), and convulsion 92 (41.4%).

In the postnatal period, vaginal bleeding 145 (65.3%) was the most frequently mentioned sign, followed by high fever 131 (59%), foul-smelling vaginal discharge 98 (44.1%), severe weakness 93 (41.9%), and difficulty breathing 83 (37.4%) (Table 4).

**Table 4:** Knowledge of obstetric danger signs mentioned by the respondents (n = 222).

(a) During pregnancy		
**Danger sign**	**Frequency (n)**	**Percent (%)**
Vaginal bleeding	144	64.9
Blurred vision	126	56.8
Swollen face/limbs	119	53.6
Severe headache	108	48.6
Hypertension	75	33.8
Convulsions	73	32.9
Persistent nausea /vomiting	71	32
Malpresentation/position	71	32
Reduced/absent fetal movement	68	30.6
Severe abdominal cramp	66	29.7
Leakage of amniotic fluid	46	20.7
**(b) During childbirth**		
**Danger sign**	**Frequency (n)**	**Percent (%)**
Prolonged labor	150	67.6
Vaginal bleeding	149	67.1
Retained placenta	115	51.8
Hypertension	96	43.2
Convulsions	92	41.4
Cessation of labor pain	81	36.5
High fever	80	36
Swollen face/limbs	74	33.3
Severe headache	71	32
Malpresentation/position	59	26.6
Blurred vision	52	23.4
Severe abdominal cramps	46	20.7
**(c) During postnatal period**		
**anger sign**	**Frequency (n)**	**Percent (%)**
Vaginal bleeding	145	65.3
High fever	131	59
Foul-smelling vaginal discharge	98	44.1
Severe weakness	93	41.9
Difficulty breathing	83	37.4
Swollen face/limbs	73	32.9
Severe headache	61	27.6
Unconsciousness	58	26.1
Uterine prolapse	50	22.5
Blurred vision	48	21.6
Convulsions	45	20.3

**Fig 1:**
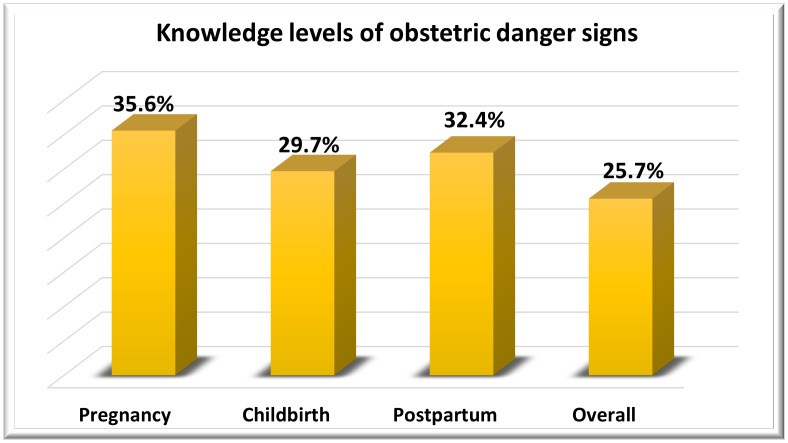
Knowledge of obstetric danger signs during pregnancy, childbirth, and postpartum periods (n = 222).

Overall, only 79 respondents (35.6%) demonstrated adequate knowledge of obstetric danger signs during pregnancy. Knowledge of danger signs was lower during childbirth, with 66 respondents (29.7%) identified as having good knowledge, and during the postnatal period, with 72 respondents (32.4%). When considering knowledge across all the three periods, only 57 respondents (25.7%) demonstrated adequate overall knowledge of obstetric danger signs (Fig 1). Notably, 35 participants (15.8%) were unable to mention even one of the three key danger signs across all three phases.

### Factors associated with knowledge of obstetric danger signs

Bivariate and multivariate logistic regression analyses were conducted to identify factors associated with knowledge of key obstetric danger signs during pregnancy. In the bivariate analysis, women’s educational status, access to media, place of delivery, decision-making autonomy, and receipt of health education during ANC were significantly associated. In the multivariate analysis, three factors remained significant predictors: women’s education level, access to media, and place of delivery.

Women with secondary education or higher were 3.6 times more likely to have good knowledge compared to those with primary education or less (AOR = 3.6, 95% CI: 1.8–7.3, p < 0.001). Access to mainstream or social media was also significant; women with such access were 2.6 times more likely to have good knowledge (AOR = 2.6, 95% CI: 1.2–5.6, p = 0.016). Similarly, women who delivered their last child in a health facility were 3.4 times more likely to have good knowledge compared to those who delivered at home (AOR = 3.4, 95% CI: 1.6–7.2, p = 0.001) (Table 5).

**Table 5:** Factors associated with knowledge of obstetric danger signs (n = 222).

Variable	Category	Good knowledge n (%)	Poor knowledge n (%)	COR (95% CI)	AOR (95% CI)
Education	Primary or less	25 (31.6%)	103 (72%)	1	1
	Secondary or more	54 (68.4%)	40 (28%)	5.5 (3.0–10.1) p < 0.001	3.6 (1.8–7.3) p < 0.001
Access to media	Yes	66 (83.5%)	76 (53.1)	4.5 (2.3–8.82) p < 0.001	2.6 (1.2–5.6) p = 0.016
	No	13 (16.5%)	67 (46.9%)	1	1
Place of last delivery	Home	13 (17.1%)	68 (51.1%)	1	1
	Hospital	63 (82.9%)	65 (48.9%)	5.0 (2.5–10) p < 0.001	3.4 (1.6–7.2) p < 0.001

## Discussion

This study assessed maternal knowledge of obstetric danger signs among women attending antenatal care in Hargeisa, Somaliland, and identified key socio-demographic and service-related predictors.

The findings revealed that knowledge of obstetric danger signs during pregnancy was low, with only slightly more than one-third of women (35.6%) classified as having good knowledge. This indicates a significant proportion of women may lack necessary awareness to recognize early warning signs of complications, potentially delaying timely health-seeking behavior and increasing the risk of adverse maternal outcomes. The relatively low knowledge observed in this study may be attributed to limited health education coverage, cultural norms influencing communication about reproductive health, and gaps in community-based health promotion. In line with this finding, studies conducted in Debre Tabor, Ethiopia (39%) [15]; Jigjiga, Ethiopia (37.9%) [16]; and Zambia (35%) [8] also reported comparable insufficient awareness of obstetric danger signs despite the implementation of maternal health programs. Similarly, lower levels of knowledge were reported in Dire Dawa, Ethiopia (24.1%) [4] and Southern Nigeria (24.9%) [17].

The findings revealed considerable gaps in awareness, particularly concerning pregnancy-related complications. While a proportion of respondents were able to identify one or more key danger signs, a considerable number of women were unable to mention any, indicating a critical gap in basic knowledge. This gap in knowledge raises serious public health concerns, as unawareness of warning symptoms may delay timely health-seeking behavior and increase the risk of adverse maternal outcomes. Additionally, these women would be at higher risk of failing to seek emergency care promptly if complications arise. Similar patterns have been documented in resource-limited settings, such as in Bhutan [18] and in a nationwide study conducted in the Democratic Republic of Congo (DRC) [19], where women often rely on traditional beliefs, family influence, or wait until complications worsen before deciding to seek care. The lack of knowledge among these women reflects missed opportunities for health education during routine antenatal visits, which are intended to serve not only as check-ups but also as platforms for prevention counselling.

Despite moderate ANC utilization, the number of women achieving the recommended four visits was optimal. Furthermore, in our study, spousal communication regarding maternal health was notably low (38.3%), a factor that may hinder timely decision-making during emergencies, in contrast to male involvement promoting positive maternal health service utilization. A systematic review and meta-analysis from low- and middle-income countries likewise positively identified male involvement as a positive factor to maternal health-seeking behavior including knowledge of obstetric danger signs [20].

Vaginal bleeding emerged as the most commonly recognized danger sign across pregnancy, childbirth, and the postpartum period, suggesting that more visible symptoms are more easily recognized. In contrast, danger signs such as convulsion and blurred vision were less commonly mentioned, highlighting knowledge gaps in recognizing less visible but equally life-threatening complications like pre-eclampsia and eclampsia. This finding aligns with studies in Shashamene, Ethiopia [7], Cameroon [21], eastern DRC [22], and Rwanda [23], as well as with a systematic review from developing countries [24], where bleeding was consistently the most frequently recognized danger sign.

Educational status appeared to play an important role in knowledge levels, as women with secondary or higher education were more likely to correctly identify danger signs than their less-educated counterparts with little or no schooling. This reflects the influence of education on awareness levels, underscoring the critical role of female education in enhancing maternal health outcomes. This finding aligns with evidence from other studies conducted in Bahir Dar, Ethiopia [14], Cameroon [21], India [25], Indonesia [26], southern Nigeria [17], and Tanzania [27], where literacy was positively associated with maternal knowledge and health seeking behavior.

Access to and use of media was one of the factors associated with good danger signs knowledge, suggesting that health promotion through radio, television, and social media platforms can play a crucial role in improving maternal awareness. These results highlight the critical role of accessible information channels in shaping maternal health outcomes, particularly in settings where formal education levels may be relatively low. Such media exposures and campaigns not only increased awareness of obstetric danger signs but also encouraged greater utilization of community-based interventions to bridge knowledge gaps and promote positive health-seeking behavior. This finding is consistent with studies in Jigjiga, Ethiopia [16], and Indonesia [26], where mass media health education was proven effective in enhancing health literacy among women of reproductive age.

Institutional delivery was another strong predictor of knowledge, as women who delivered in health facilities were more likely to recall danger signs, possibly due to counselling received during delivery and postnatal care. This suggests that exposure to skilled care environments enhanced maternal awareness through repeated counselling opportunities. Moreover, it underlines the role of health facilities as important platforms for reinforcing health education beyond routine antenatal services. This finding is consistent with WHO recommendations emphasizing skilled birth attendance as a key avenue for maternal health education [28]. Similarly, evidence from a systematic review in developing countries [24], and a study conducted in Zambia [8] support this association, stressing the role of facility-based care in improving women’s awareness.

Overall, the results suggest that while some progress has been made, substantial gaps remain in maternal knowledge, particularly concerning less obvious danger signs and across the pregnancy, childbirth and postnatal periods.

### Strengths and limitations

The study provides context-specific evidence on maternal knowledge of obstetric danger signs, which is essential for designing targeted interventions. In addition, the use of a structured questionnaire allowed for systematic assessment of knowledge across multiple domains. However, the following limitations should be considered. The cross-sectional design precludes establishing cause-and-effect relationships. Self-reported knowledge may be subject to recall or social desirability bias. Furthermore, the study was limited to women attending health facilities, which may not represent those who do not access ANC services and who might have even lower awareness. Despite its limitations, the study provides valuable evidence for public health by identifying gaps in maternal knowledge of obstetric danger signs.

### Conclusion

This study demonstrated that knowledge of obstetric danger signs among pregnant women in Hargeisa is low, with only about one-third of the respondents having good knowledge about danger signs during pregnancy and barely one-quarter demonstrating overall adequate knowledge. It highlights critical gaps in maternal knowledge, with a particular concerning proportion of women unable to identify any key warning signs. Vaginal bleeding was the most commonly recognized danger sign, but awareness of other critical signs was limited. Maternal education, access to media, and institutional delivery were identified as key determinants of knowledge. Therefore, designing enhanced and targeted health education programs, and facility-based interventions can help bridge the critical gaps.

## Supporting information

S1 File SPSS_Knowledge_ODS_PLOS.sav. (SAV)

S2 File Knowledge_ODS_PLOS.xlsx (XLSX)
