# Supplementary material for: Maternal knowledge of obstetric danger signs and related factors among antenatal care attendees in Hargeisa, Somaliland: A cross-sectional analysis
Source: PLoS One. 2026 Jul 15;21(7):e0353778. doi: 10.1371/journal.pone.0353778 (PMC13372116; doi:10.1371/journal.pone.0353778)
Supplement: Attachment — Submitted filename: PONE-D-25-51252_R1 (1).pdf [file pone.0353778.s005.pdf]

PLOS One

Maternal Knowledge of Obstetric Danger Signs and Related Factors among Antenatal Care Attendees in Hargeisa, Somaliland: A Cross-Sectional Analysis

--Manuscript Draft--

**Manuscript Number:**	PONE-D-25-51252R1
**Article Type:**	Research Article
**Full Title:**	Maternal Knowledge of Obstetric Danger Signs and Related Factors among Antenatal Care Attendees in Hargeisa, Somaliland: A Cross-Sectional Analysis
**Short Title:**	Knowledge of Obstetric Danger Signs
**Corresponding Author:**	Abdeta Muktar Ahmed<br>Addis Ababa Medical University College Hargeisa Campus<br>Hargeisa, Somaliland SOMALIA
**Keywords:**	obstetric danger signs, maternal knowledge, antenatal care, pregnancy complications, Hargeisa, Somaliland
**Abstract:**	Background: Maternal mortality remains a major global health challenge, disproportionately affecting low- and middle-income countries. Despite international efforts, nearly 95% of maternal deaths occur in these regions, with Sub-Saharan Africa bearing the highest burden. Limited knowledge of obstetric danger signs is a key factor contributing to delays in seeking care, which in turn increases the risk of preventable maternal complications. Evidence on maternal awareness in Somaliland is scarce.<br>Objective: This study aimed to assess the level of knowledge of obstetric danger signs and associated factors among pregnant women attending antenatal care in Hargeisa, Somaliland.<br>Methods: A facility-based cross-sectional study was conducted from July 2 to August 4, 2022, among pregnant women attending antenatal care services. A total of 222 participants were selected using a systematic random sampling technique from eight maternal and child health centers. Data were collected through face-to-face interviews using a pretested and structured questionnaire and analyzed with SPSS version 25. Bivariate and multivariate logistic regression was carried out.<br>Results: About 35.6% had a good knowledge specifically about pregnancy related obstetric danger signs. Only 57 women (25.7%) demonstrated overall good knowledge of obstetric danger signs across pregnancy, childbirth, and the postpartum periods. Knowledge was highest for vaginal bleeding (65% - 67%), while less visible complications such as convulsions and blurred vision were less frequently mentioned. Multivariate analysis revealed that secondary level education or higher (AOR = 3.6, 95% CI: 1.8-7.3), access to and usage of media (AOR = 2.6, 95% CI: 1.2-5.6), and institutional delivery (AOR = 3.4, 95% CI: 1.6-7.2), were significant predictors of knowledge.<br>Conclusion: Maternal knowledge of obstetric danger signs in Hargeisa was low. Targeted health education, broader use of mass media, and promoting institutional delivery are critical strategies to improve and reduce preventable maternal mortality.
**Order of Authors:**	Abdeta Muktar Ahmed
	Mohamed Abdilahi Ahmed
	Abdulkadir Mohammed Nuh
	Hamse Khalif Hassan
**Response to Reviewers:**	The comments, queries and suggestions were addressed and uploaded to the system.
**Additional Information:**	
**Question**	**Response**
**Financial Disclosure**<br><br>Enter a financial disclosure statement that describes the sources of funding for the	The author(s) received no specific funding for this work.

work included in this submission. Review the submission guidelines for detailed requirements. View published research articles from *PLOS ONE* for specific examples.<br><br>This statement is required for submission and **will appear in the published article** if the submission is accepted. Please make sure it is accurate.<br><br>**Funded studies**<br>Enter a statement with the following details:<br>• Initials of the authors who received each award<br>• Grant numbers awarded to each author<br>• The full name of each funder<br>• URL of each funder website<br>• Did the sponsors or funders play any role in the study design, data collection and analysis, decision to publish, or preparation of the manuscript?<br><br>Did you receive funding for this work?	
**Competing Interests**<br><br>Use the instructions below to enter a competing interest statement for this submission. On behalf of all authors, disclose any competing interests that could be perceived to bias this work—acknowledging all financial support and any other relevant financial or non-financial competing interests.<br><br>This statement is required for submission and **will appear in the published article** if the submission is accepted. Please make sure it is accurate and that any funding sources listed in your Funding Information later in the submission form are also declared in your Financial Disclosure statement.<br><br>View published research articles from *PLOS ONE* for specific examples.	The authors have declared that no competing interests exist.

Powered by Editorial Manager® and ProduXion Manager® from Aries Systems Corporation

**NO authors have competing interests**<br><br>Enter: *The authors have declared that no competing interests exist.*<br><br>**Authors with competing interests**<br><br>Enter competing interest details beginning with this statement:<br><br>*I have read the journal's policy and the authors of this manuscript have the following competing interests: [insert competing interests here]*<br><br>* typeset	
**Data Availability**<br><br>Authors are required to make all data underlying the findings described fully available, without restriction, and from the time of publication. PLOS allows rare exceptions to address legal and ethical concerns. See the PLOS Data Policy and FAQ for detailed information.<br><br>A Data Availability Statement describing where the data can be found is required at submission. Your answers to this question constitute the Data Availability Statement and **will be published in the article**, if accepted.<br><br>**Important:** Stating 'data available on request from the author' is not sufficient. If your data are only available upon request, select 'No' for the first question and explain your exceptional situation in the text box.<br><br>Do the authors confirm that all data underlying the findings described in their manuscript are fully available without restriction?	Yes - all data are fully available without restriction
Additional data availability information:	
**Ethics Statement**<br><br>Enter an ethics statement for this	Ethical approval for the study was obtained from the Research Ethics Committee of Addis Ababa Medical University College, Hargeisa campus (Ref. No: AAMUCHC/REC/87/22). The purpose of the study was explained to all participants,

submission. This statement is required if the study involved:<br><br>• Human participants<br>• Human specimens or tissue<br>• Vertebrate animals or cephalopods<br>• Vertebrate embryos or tissues<br>• Field research<br><br>Write "N/A" if the submission does not require an ethics statement.<br><br>General guidance is provided below. Consult the submission guidelines for detailed instructions. **Make sure that all information entered here is included in the Methods section of the manuscript.**	and written informed consent was obtained prior to data collection. Participation was voluntary, and respondents were assured that their information would be used solely for research purpose and kept confidential. Names and personal identifiers were not recorded to preserve anonymity.

Powered by Editorial Manager® and ProduXion Manager® from Aries Systems Corporation

**Format for specific study types**<br><br>**Human Subject Research (involving human participants and/or tissue)**<br>• Give the name of the institutional review board or ethics committee that approved the study<br>• Include the approval number and/or a statement indicating approval of this research<br>• Indicate the form of consent obtained (written/oral) or the reason that consent was not obtained (e.g. the data were analyzed anonymously)<br><br>**Animal Research (involving vertebrate animals, embryos or tissues)**<br>• Provide the name of the Institutional Animal Care and Use Committee (IACUC) or other relevant ethics board that reviewed the study protocol, and indicate whether they approved this research or granted a formal waiver of ethical approval<br>• Include an approval number if one was obtained<br>• If the study involved *non-human primates*, add *additional details* about animal welfare and steps taken to ameliorate suffering<br>• If anesthesia, euthanasia, or any kind of animal sacrifice is part of the study, include briefly which substances and/or methods were applied<br><br>**Field Research**<br><br>Include the following details if this study involves the collection of plant, animal, or other materials from a natural setting:<br>• Field permit number<br>• Name of the institution or relevant body that granted permission	
Data Availability<br><br>Before publication, Authors are required to make fully available and without restriction all data underlying their findings. Sharing the data underlying your findings increases the visibility, credibility, and impact of your work, while supporting transparency and future scientific discovery. Please see our PLOS Data Policy page for detailed information on this policy.</p><br>A Data Availability	All relevant data are within the manuscript and its Supporting Information files.

Statement, detailing where the data can be accessed, is required at first submission. Insert your Data Availability Statement in the box below.</p><br><br>Please see the data reporting of our submission guidelines for instructions on what you need to include in your Data Availability Statement. Examples of acceptable Data Availability Statements are included here for reference:</p><br> <br> - The minimal data set [and accompanying code, where generated] is available at [repository name] via [DOI hyperlink] <br> - No data was generated by this study. The following existing data sources were used: [dataset name] from [dataset location] available via [web address or DOI hyperlink]. <br> - For Study Protocols: No datasets were generated or analysed during the current study. All relevant data from this study will be made available upon study completion. <br> <br><br><i>PLOS allows rare exemptions to address legal and ethical concerns. If you have legal or ethical restrictions, please use the box below to detail these in full sentences for the Journal team to consider.</i></p><br><br>This statement will be typeset if the manuscript is accepted for publication.</p>	

Powered by Editorial Manager® and ProduXion Manager® from Aries Systems Corporation

Manuscript Click here to access/download;Manuscript;Manuscript.docx

Maternal Knowledge of Obstetric Danger Signs and Related Factors among Antenatal Care Attendees in Hargeisa, Somaliland: A Cross-Sectional Analysis

Abdeta Muktar Ahmed [1*], Mohamed Abdilahi Ahmed [1], Abdulkadir Mohammed Nuh [2], Hamse Khalif Hassan [1,3]

[1] Addis Ababa Medical University College, Hargeisa Campus, Department of Public Health, Hargeisa, Somaliland

[2] Addis Ababa Medical University College, Hargeisa Campus, Department of Nutrition, Hargeisa, Somaliland

[3] International Health program, College of Medicine, National Yang Ming Chiao Tung University (NYCU), Taipei, Taiwan

* Corresponding author

E-mail: abdetamuk16@gmail.com

Abstract

Background: Maternal mortality remains a major global health challenge, disproportionately affecting low- and middle-income countries. Despite international efforts, nearly 95% of maternal deaths occur in these regions, with Sub-Saharan Africa bearing the highest burden. Limited knowledge of obstetric danger signs is a key factor contributing to delays in seeking care, which in turn increases the risk of preventable maternal complications. Evidence on maternal awareness in Somaliland is scarce.

Objective: This study aimed to assess the level of knowledge of obstetric danger signs and associated factors among pregnant women attending antenatal care in Hargeisa, Somaliland.

Methods: A facility-based cross-sectional study was conducted from July 2 to August 4, 2022, among pregnant women attending antenatal care services. A total of 222 participants were selected using a systematic random sampling technique from eight maternal and child health centers. Data were collected through face-to-face interviews using a pretested and structured questionnaire and analyzed with SPSS version 25. Bivariate and multivariate logistic regression was carried out.

Results: About 35.6% had a good knowledge specifically about pregnancy related obstetric danger signs. Only 57 women (25.7%) demonstrated overall good knowledge of obstetric danger signs across pregnancy, childbirth, and the postpartum periods. Knowledge was highest for vaginal bleeding (65% - 67%), while less visible complications such as convulsions and blurred vision were less frequently mentioned. Multivariate analysis revealed that secondary level education or higher (AOR = 3.6, 95% CI: 1.8-7.3), access to and usage of media (AOR = 2.6,

95% CI: 1.2-5.6), and institutional delivery (AOR = 3.4, 95% CI: 1.6-7.2), were significant predictors of knowledge.

Conclusion: Maternal knowledge of obstetric danger signs in Hargeisa was low. Targeted health education, broader use of mass media, and promoting institutional delivery are critical strategies to improve and reduce preventable maternal mortality.

Key words: obstetric danger signs, maternal knowledge, antenatal care, pregnancy complications, Hargeisa, Somaliland

Background

Maternal mortality remains a pressing global issue. In 2023, more than 700 women lost their lives to pregnancy-related and childbirth-related avoidable causes every day, or roughly one woman every two minutes [1]. Despite international efforts to address it, maternal mortality is still a serious global health concern. The startling truth is that the majority of maternal fatalities are avoidable and result from severe health disparities that occur both within and between nations and worldwide. These disparities are significant. Nearly 95% of all maternal deaths take place in low- and middle-income nations, with Sub-Saharan Africa bearing the highest risk, accounting for 70% of all maternal deaths. The 2030 United Nations Sustainable Development Goal (SDG), which aims to lower the rate of maternal fatalities to fewer than 70 per 100,000 live births, is still significantly above the current global rate of maternal deaths (223 per 100,000 live births) [2-3].

Even though, an estimated 260,000 maternal deaths, or about 700 deaths per day, occurred globally in 2023, maternal mortality has decreased by 40% since 2000, which is indicative of ongoing international efforts to provide access to basic healthcare services. No region was designated as having a very high maternal mortality ratio (MMR), and no country was predicted to have an exceptionally high MMR for the first time [1].

One of the main causes of maternal mortality in the underdeveloped world is women's, families', and birth attendants' lack of awareness about the obstetric warning signs [4]. This challenge can be better understood within the framework of the Three Delays Model, which highlights delays in (a) deciding to seek care, (b) reaching a health facility, and (c) receiving appropriate care upon arrival [5]. In this context, inadequate knowledge of obstetric danger signs primarily contributes

to the first delay, as women and their families may fail to recognize life-threatening complications and seek timely medical attention. These deaths can be decreased by educating women about the warning signs of obstetric dangers, encouraging them to seek medical attention when necessary, and counselling them on these obstetric danger signs, as well as the right management of the complications [4].

Obstetric danger signs are warning indications that could point to potentially life-threatening conditions during pregnancy, labor, or the postpartum phases. Approximately 75% of maternal deaths during these three phases are caused by obstetric danger signs, which include vaginal bleeding, blurred vision, lack of fetal movement, a gush of fluid from the vagina, high fever, retained placenta, foul-smelling discharge, convulsions, and severe headaches. Because mothers and family members may quickly recognize these signs without need for a complex professional diagnosis, raising maternal knowledge is a crucial approach in minimizing care-seeking delays [6]. Pregnant women who notice warning symptoms and seek prompt obstetric care could reduce maternal mortality from complications connected to childbirth in low-income countries. One of the most frequent reasons why people fail to detect complications when they arise and put off seeking care is a lack of warning signs [7]. Poor maternal and newborn outcomes result from pregnant women's lack of awareness of obstetric danger signs, which is a major cause in delays in seeking care. Designing focused treatments that raise awareness, encourage prompt care-seeking behavior, and improve birth preparedness requires an understanding of the factors influencing pregnant women's knowledge of obstetric danger signs [8].

According to the 2020 Somaliland Health and Demographic Survey (SLHDS), the country experiences an estimated 396 maternal deaths for every 100,000 live births. Furthermore, only 47% of pregnant women reported attending at least one antenatal care (ANC) visit, and merely

33% delivered with the assistance of skilled birth attendant [9]. Although Somaliland’s maternal mortality ratio has shown a declining trend and is not among the highest in the region, it still remains far from achieving the SDG target of reducing maternal mortality to 70 per 100,000 live births by 2030.

To date, no studies have been conducted in Hargeisa, Somaliland, to assess the level of knowledge regarding obstetric danger signs and their associated factors. Understanding this knowledge gap is crucial, as improving awareness of obstetric danger signs can inform strategies to enhance maternal and health outcomes and reduce maternal mortality. Therefore, the present study aimed to evaluate the level of knowledge of obstetric danger signs and to identify the associated factors among pregnant women attending antenatal care (ANC) services in selected health institutions in Hargeisa, Somaliland.

Materials and Methods

Study design and area

A facility-based cross-sectional study was conducted in Hargeisa, the capital of Somaliland, from July 2 to August 4, 2022. Somaliland, a self-declared autonomous state in the horn of Africa that unilaterally separated from Somalia in 1991, had an estimated population of 6.2 million [10]. Hargeisa, its capital, is home to approximately 1.2 million people [11].

Study population

The source population consisted of pregnant women attending ANC services at health institutions in Hargeisa during the study period.

Inclusion criteria: All pregnant women attending antenatal care services in the selected health institutions during the study period who were physically and mentally able and willing to participate.

Exclusion criteria: Pregnant women who were critically ill at the time of data collection, defined as those experiencing severe medical or obstetric conditions that impaired their ability to communicate or safely participate in an interview.

The sample size was calculated using a single population proportion formula:

$n = (Z^2 \text{ x } p \text{ x } (1\text{-}p)) / d^2$

Where Z = 1.96 (95% confidence level), p = 0.155 (15.5% prevalence of knowledge of obstetric danger signs from a previous study [12]), and 5% margin of error.

$$n = (1.96^2 \times 0.155 \times 0.845) / (0.05^2) \approx 201$$

After adding a 10% non-response rate, the final sample size became 222.

Sampling procedure

Eight centers were selected through simple random sampling from the total of 17 Maternal and Child Health (MCH) centers to ensure representativeness and minimize selection bias.

The selected health facilities were public Maternal and Child Health (MCH) centers that provide comprehensive primary healthcare services, including antenatal care, delivery services, postnatal care, and child health services. In addition, routine maternal services including health education, nutritional counselling, basic laboratory investigations, and clinical examinations are offered. The facilities are staffed by trained healthcare professionals, including midwives and nurses. As government-run institutions, they follow standardized service delivery protocols, ensuring relatively uniform care across facilities. These MCH centers are widely distributed throughout Hargeisa city, making them highly accessible and commonly utilized by the population.

Proportional allocation was applied to distribute the study units across the selected facilities, based on the number of ANC attendees recorded in the month preceding the survey. The sampling interval (K) was calculated as about three, meaning every third eligible woman attending ANC was invited to participate until the allocated quota for each facility was reached.

Research Instrument and measurement

Data were collected using pretested, structured, interviewer-administered questionnaire adapted from the Maternal and Newborn Health Program of JHPIEGO, an affiliate of Johns Hopkins University, and other related articles [13-14]. The tool comprised four sections: (1)

Sociodemographic characteristics, (2) Reproductive and obstetric history, (3) Service utilization, and (4) Knowledge of obstetric danger signs. The questionnaire was initially prepared in English, translated into Somali, and subsequently back-translated into English to ensure consistency and accuracy.

Women's knowledge of obstetric danger signs was assessed by asking participants to spontaneously mention danger signs that may occur during pregnancy, childbirth, and the postpartum period, which were categorized into three groups. Knowledge of pregnancy danger signs (the study's dependent variable) was defined as correctly identifying at least three key signs, including vaginal bleeding, swollen hands/face, and blurred vision. Knowledge of childbirth/labor danger signs was defined as mentioning at least three major signs, namely vaginal bleeding, prolonged labor, convulsions, or retained placenta. For the postpartum period, women were regarded as having good knowledge if they identified at least three major signs, including vaginal bleeding after delivery, loss of consciousness, and postpartum fever. Overall, a woman was considered as having good knowledge on obstetric danger signs if she was able to mention at least three key signs across each of the three phases, without prompting, in line with the JHPIEGO maternal health framework [13, 14]. Those who mention fewer than three key danger signs were categorized as having poor knowledge. Hence, for analysis, the dependent variables were dichotomized into "**Good knowledge**" and "**Poor knowledge**." This classification approach has been widely used in similar studies assessing maternal knowledge of obstetric danger signs in low- and middle-income settings.

Data collection procedures

Data were collected through face-to-face interview conducted by six trained BSc Nursing students. Two experienced supervisors oversaw the process. Prior to data collection, the questionnaire was pretested on 22 pregnant women at an MCH center outside the study area. Based on pretest findings, adjustments were made to improve clarity and accuracy. Training was provided to data collectors and supervisors on the objectives of the study, the data collection tool, ethical considerations, and procedures to ensure confidentiality.

Data processing and analysis

Completed questionnaires were checked daily for completeness and consistency. Data were cleaned, coded, and entered into SPSS version 25 for analysis. Descriptive statistics were used to summarize the data. Bivariate logistic regression was first performed, and variables with a p-value < 0.2 were considered candidates for multivariate analysis. Subsequently, multivariate logistic regression was carried out to examine associations between independent variables and the outcome variable while controlling for potential confounding factors. Adjusted odds ratios (AOR) with 95% confidence interval (CI) were computed to determine the strength of associations. Statistical significance was declared at $p<0.05$.

Ethical considerations

Ethical approval for the study was obtained from the Research Ethics Committee of Addis Ababa Medical University College, Hargeisa campus (Ref. No: AAMUCHC/REC/87/22). The purpose of the study was explained to all participants, and written informed consent was obtained prior to data collection. Participation was voluntary, and respondents were assured that their information would be used solely for research purpose and kept confidential. Names and personal identifiers were not recorded to preserve anonymity.

Result

Socio-demographic characteristics

A total of 222 pregnant women participated in the study, yielding a response rate of 100%. The mean age of the respondents was 28.3 years (±6.5 SD), with a median age of 28.5 years, ranging from 17 to 43 years. More than half of the respondents (55.4%) were between 25 and 36 years of age. Most participants 201 (90.5%) were currently married. Regarding educational attainment, 75 (33.8%) had no formal education, 53 (23.9%) had completed primary education, 38 (17.1%) had secondary education, and 56 (25.2%) attained higher education. By contrast, more than half of their spouses 121 (54.5%) had higher education, while only 59 (26.6%) had primary or no formal education. In terms of occupation, the majority of respondents 153 (68.9%) were merchants, 49 (22.1%) were housewives/unemployed, and 20 (9%) were formally employed. Among spouses, 149 (67.1%) were employed, 62 (27.9%) were merchants, and 11 (5%) were unemployed. The mean monthly household income was 561 USD. A large proportion 152 (68.5%) reported monthly incomes of ≤500 USD. Additionally, 142 respondents (64%) had access to mainstream or social media (Table 1).

Table 1. Socio-demographic characteristics of the respondents (n=222)

Variables	Category	Frequency (n)	Percentage (%)
Age (years)	≤24	70	31.5
	25-36	123	55.4
	≥37	29	13.1
Marital status	Currently married	201	90.5
	Widowed/Divorced	21	9.5

Educational status (women)	No formal education	75	33.8
	Primary	53	23.9
	Secondary	38	17.1
	Higher education	56	25.2
Educational status (spouse)	No formal education	36	16.2
	Primary	23	10.4
	Secondary	42	18.9
	Higher education	121	54.5
Occupation (women)	Merchant	153	68.9
	Housewife/Unemployed	49	22.1
	Employed	20	9
Occupation (Spouse)	Employed	149	67.1
	Merchant	62	27.9
	Unemployed	11	5
Monthly family income (USD)	≤500	152	68.5
	501-1000	30	13.5
	>1000	40	18
Access to media	Yes	142	64
	No	80	36

Obstetric characteristics

The age at first marriage ranged from 14 to 28 years, with a mean of 18.9 years. Half of the respondents 111 (50%) married between the age of 14-18 years. The age at first pregnancy ranged from 16 to 30 years, with a mean of 20 years; more than half 119 (53.6%) conceived between 19-24 years. At the time of data collection, nearly half of the respondents 102 (45.9%) were in their third trimester.

Number of pregnancies ranged from 1 to 11, with a mean of 4.5, of which 95 (42.8%) had experienced 4-6 pregnancies. Meanwhile, 136 participants (61.3%) reported having up to three births. Regarding pregnancy intention, 144 (64.9%) stated their current pregnancy was planned, while 78 (35.1%) described otherwise. The majority of participants, 178 (80.2%) reported not experiencing any obstetric danger signs during their current pregnancy (Table 2).

Table 2. Obstetric variables of the study participants (n=222)

**Variables**	**Category**	**Frequency (n)**	**Percent (%)**
Age at first marriage (years)	14-18	111	50
	19-23	92	41.4
	≥24	19	8.6
Age at first pregnancy (years)	16-19	108	48.6
	20-23	84	37.8
	≥24	30	13.5
Trimester (Current pregnancy)	First	54	24.5
	Second	66	29.7
	Third	102	45.9
Number of pregnancies	≤3	83	37.4
	4-6	95	42.8
	>6	44	19.8
Number of births	≤3	136	61.3
	4-6	62	27.9
	>6	24	10.8
Current pregnancy intended	Yes	144	64.9
	No	78	35.1
History of danger signs	Yes	44	19.8
	No	178	80.2

Maternal Health Service Utilization

With respect to ANC visits during the current pregnancy, 63 women (28.4%) had attended only once, while 66 (29.7%), 55 (24.8%), and 38 (17.2%) had attended two, three, and four or more visits, respectively. More than two-thirds 158 (71.2%) had received health education about obstetric danger signs during ANC visits, whereas 64 (28.8%) had not.

Among 209 respondents with previous childbirth experience, 128 (61.2%) delivered their last baby in a health institution, while 81 (38.8%) delivered at home. The majority 187 (89.5%) reported spontaneous vaginal delivery, followed by 15 (7.2%) instrumental delivery, and 7 (3.3%) cesarean section.

The reported travel time to the nearest health facility ranged from 1 to 120 minutes, with a mean of 33 minutes. More than half, 130 (58.6%) lived within a 30-minutes walking distance. Regarding decision-making autonomy, 116 (52.3%) reported making decisions independently, 82 (36.9%) jointly with their partner, and 24 (10.8%) reported that decisions were solely by their partner. Only 85 (38.3%) discussed maternal health service utilization with their spouses, while the majority, 137 (61.7%) did not (Table 3).

Table 3. Maternal health service utilization (n=222)

Variables	Category	Frequency (n)	Percentage (%)
ANC visits (current pregnancy)	1-3	184	82.9
	≥4	38	17.2
Received health education on danger signs	Yes	158	71.2
	No	64	28.8
Place of last delivery (n=209)	Home	81	38.8
	Health institution	128	61.2

Mode of delivery (n=209)	Spontaneous vaginal delivery (SVD)	187	89.5
	Instrumental Delivery	15	7.2
	Cesarean section	7	3.3
Time to nearest health facility (walk)	≤30 min	130	58.6
	>30 min	92	41.4
Decision making power	Self	116	52.3
	Joint (both)	82	36.9
	Partner only	24	10.8
Discussion with spouse on maternal services	Yes	85	38.3
	No	135	61.7

Knowledge of obstetric Danger Signs

Respondents were asked to list key obstetric danger signs spontaneously. During pregnancy, the most commonly mentioned signs were vaginal bleeding by 144 participants (64.9%), blurred vision 126 (56.8%), swollen face and limbs 119 (53.6%), severe headache 108 (48.6%), hypertension 75 (33.8%), and convulsion 73 (32.9%).

During childbirth, prolonged labor by 150 respondents (67.6%) and vaginal bleeding by 149 (67.1%) were the most frequently mentioned danger signs, followed by retained placenta 115 (51.8%), hypertension 96 (43.2%), and convulsion 92 (41.4%).

In the postnatal period, vaginal bleeding 145 (65.3%) was the most frequently mentioned sign, followed by high fever 131 (59%), foul-smelling vaginal discharge 98 (44.1%), severe weakness 93 (41.9%), and difficulty breathing 83 (37.4%) (Table 4).

Table 4. Knowledge of obstetric danger signs mentioned by the respondents (n=222)

(a) During pregnancy

Danger sign	Frequency (n)	Percent (%)
Vaginal bleeding	144	64.9
Blurred vision	126	56.8
Swollen face/limbs	119	53.6
Severe headache	108	48.6
Hypertension	75	33.8
Convulsions	73	32.9
Persistent nausea /vomiting	71	32
Malpresentation/position	71	32
Reduced/absent fetal movement	68	30.6
Severe abdominal cramp	66	29.7
Leakage of amniotic fluid	46	20.7

(b) During childbirth

Danger sign	Frequency (n)	Percent (%)
Prolonged labor	150	67.6
Vaginal bleeding	149	67.1
Retained placenta	115	51.8
Hypertension	96	43.2
Convulsions	92	41.4
Cessation of labor pain	81	36.5
High fever	80	36
Swollen face/limbs	74	33.3
Severe headache	71	32
Malpresentation/position	59	26.6
Blurred vision	52	23.4
Severe abdominal cramps	46	20.7

(c) During postnatal period

**Danger sign**	**Frequency (n)**	**Percent (%)**
Vaginal bleeding	145	65.3
High fever	131	59
Foul-smelling vaginal discharge	98	44.1
Severe weakness	93	41.9
Difficulty breathing	83	37.4
Swollen face/limbs	73	32.9
Severe headache	61	27.6
Unconsciousness	58	26.1
Uterine prolapse	50	22.5
Blurred vision	48	21.6
Convulsions	45	20.3

Overall, only 79 respondents (35.6%) demonstrated adequate knowledge of obstetric danger signs during pregnancy. Knowledge of danger signs was lower during childbirth, with 66 respondents (29.7%) identified as having good knowledge, and during the postnatal period, with 72 respondents (32.4%). When considering knowledge across all the three periods, only 57 respondents (25.7%) demonstrated adequate overall knowledge of obstetric danger signs (Figure 1). Notably, 35 participants (15.8%) were unable to mention even one of the three key danger signs across all three phases.

Figure 1. Knowledge of obstetric danger signs during pregnancy, childbirth, and postpartum periods (n=222)

Factors Associated with Knowledge of Obstetric Danger Signs

Bivariate and multivariate logistic regression analyses were conducted to identify factors associated with knowledge of key obstetric danger signs during pregnancy. In the bivariate analysis, women's educational status, access to media, place of delivery, decisions-making autonomy, and receipt of health education during ANC were significantly associated. In the multivariate analysis, three factors remained significant predictors: women's education level, access to media, and place of delivery.

Women with secondary education or higher were 3.6 times more likely to have good knowledge compared to those with primary education or less (AOR = 3.6, 95% CI: 1.8-7.3, $p < 0.001$). Access to mainstream or social media was also significant; women with such access were 2.6 times more likely to have good knowledge (AOR = 2.6, 95% CI: 1.2-5.6, $p = 0.016$). Similarly, women who delivered their last child in a health facility were 3.4 times more likely to have good knowledge compared to those who delivered at home (AOR = 3.4, 95% CI: 1.6-7.2, $p = 0.001$) (Table 5).

Table 5. Factors associated with knowledge of obstetric danger signs (n=222)

Variable	Category	Good knowledge n (%)	Poor knowledge n (%)	COR (95% CI)	AOR (95% CI)
Education	Primary or less	25 (31.6%)	103 (72%)	1	1
	Secondary or more	54 (68.4%)	40 (28%)	5.5 (3.0-10.1) p<0.001	3.6 (1.8-7.3) p<0.001
Access to media	Yes	66 (83.5%)	76 (53.1)	4.5 (2.3-8.82) p<0.001	2.6 (1.2-5.6) p=0.016
	No	13 (16.5%)	67 (46.9%)	1	1
Place of last delivery	Home	13 (17.1%)	68 (51.1%)	1	1
	Hospital	63 (82.9%)	65 (48.9%)	5.0 (2.5- 10) p<0.001	3.4 (1.6-7.2) p<0.001

Discussion

This study assessed maternal knowledge of obstetric danger signs among women attending antenatal care in Hargeisa, Somaliland, and identified key socio-demographic and service-related predictors.

The findings revealed that knowledge of obstetric danger signs during pregnancy was low, with only slightly more than one-third of women (35.6%) classified as having good knowledge. This indicates a significant proportion of women may lack necessary awareness to recognize early warning signs of complications, potentially delaying timely health-seeking behavior and increasing the risk of adverse maternal outcomes. The relatively low knowledge observed in this study may be attributed to limited health education coverage, cultural norms influencing communication about reproductive health, and gaps in community-based health promotion. In line with this finding, studies conducted in Debre Tabor, Ethiopia (39%) [15]; Jigjiga, Ethiopia (37.9%) [16]; and Zambia (35%) [8] also reported comparable insufficient awareness of obstetric danger signs despite the implementation of maternal health programs. Similarly, lower levels of knowledge were reported in Dire Dawa, Ethiopia (24.1%) [4] and Southern Nigeria (24.9%) [17].

The findings revealed considerable gaps in awareness, particularly concerning pregnancy-related complications. While a proportion of respondents were able to identify one or more key danger signs, a considerable number of women were unable to mention any, indicating a critical gap in basic knowledge. This gap in knowledge raises serious public health concerns, as unawareness of warning symptoms may delay timely health-seeking behavior and increase the risk of adverse maternal outcomes. Additionally, these women would be at higher risk of failing to seek

emergency care promptly if complications arise. Similar patterns have been documented in resource-limited settings, such as in Bhutan [18] and in a nationwide study conducted in the Democratic Republic of Congo (DRC) [19], where women often rely on traditional beliefs, family influence, or wait until complications worsen before deciding to seek care. The lack of knowledge among these women reflects missed opportunities for health education during routine antenatal visits, which are intended to serve not only as check-ups but also as platforms for prevention counselling.

Despite moderate ANC utilization, the number of women achieving the recommended four visits was optimal. Furthermore, in our study, spousal communication regarding maternal health was notably low (38.3%), a factor that may hinder timely decision-making during emergencies, in contrast to male involvement promoting positive maternal health service utilization. A systematic review and meta-analysis from low- and middle-income countries likewise positively identified male involvement as a positive factor to maternal health-seeking behavior including knowledge of obstetric danger signs [20].

Vaginal bleeding emerged as the most commonly recognized danger sign across pregnancy, childbirth, and the postpartum period, suggesting that more visible symptoms are more easily recognized. In contrast, danger signs such as convulsion and blurred vision were less commonly mentioned, highlighting knowledge gaps in recognizing less visible but equally life-threatening complications like pre-eclampsia and eclampsia. This finding aligns with studies in Shashamene, Ethiopia [7], Cameroon [21], eastern DRC [22], and Rwanda [23], as well as with a systematic review from developing countries [24], where bleeding was consistently the most frequently recognized danger sign.

Educational status appeared to play an important role in knowledge levels, as women with secondary or higher education were more likely to correctly identify danger signs than their less-educated counterparts with little or no schooling. This reflects the influence of education on awareness levels, underscoring the critical role of female education in enhancing maternal health outcomes. This finding aligns with evidence from other studies conducted in Bahir Dar, Ethiopia [14], Cameroon [21], India [25], Indonesia [26], southern Nigeria [17], and Tanzania [27], where literacy was positively associated with maternal knowledge and health seeking behavior.

Access to and use of media is one of factors associated with good danger signs knowledge, suggesting that health promotion through radio, television, and social media platforms can play a crucial role in improving maternal awareness. These results highlight the critical role of accessible information channels in shaping maternal health outcomes, particularly in settings where formal educations levels may be relatively low. Such media exposures and campaigns not only increased awareness of obstetric danger signs but also encouraged greater utilization of community-based interventions to bridge knowledge gaps and promote positive health-seeking behavior. This finding is consistent with studies in Jigjiga, Ethiopia [16], and Indonesia [26], where mass media health education was proven effective in enhancing health literacy among women of reproductive age.

Institutional delivery was another strong predictor of knowledge, as women who delivered in health facilities were more likely to recall danger signs, possibly due to counselling received during delivery and postnatal care. This suggests that exposure to skilled care environments enhanced maternal awareness through repeated counselling opportunities. Moreover, it underlines the role of health facilities as important platforms for reinforcing health education beyond routine antenatal services. This finding is consistent with WHO recommendations

emphasizing skilled birth attendance as a key avenue for maternal health education [28]. Similarly, evidence from a systematic review in developing counties [24], and a study conducted in Zambia [8] support this association, stressing the role of facility-based care in improving women's awareness.

Overall, the results suggest that while some progress has been made, substantial gaps remain in maternal knowledge, particularly concerning less obvious danger signs and across the pregnancy, childbirth and postnatal periods.

Strengths and limitations

The study provides context-specific evidence on maternal knowledge of obstetric danger signs, which is essential for designing targeted interventions. In addition, the use of a structured questionnaire allowed for systematic assessment of knowledge across multiple domains. However, the following limitations should be considered. The cross-sectional design precludes establishing cause-and-effect relationships. Self-reported knowledge may be subject to recall or social desirability bias. Furthermore, the study was limited to women attending health facilities, which may not represent those who do not access ANC services and who might have even lower awareness. Despite its limitations, the study provides valuable evidence for public health by identifying gaps in maternal knowledge of obstetric danger signs.

Conclusion

This study demonstrated that knowledge of obstetric danger signs among pregnant women in Hargeisa is low, with only about one-third of the respondents having good knowledge about danger signs during pregnancy and barely one-quarter demonstrating overall adequate knowledge. It highlights critical gaps in maternal knowledge, with a particular concerning proportion of women unable to identify any key warning signs. Vaginal bleeding was the most commonly recognized danger sign, but awareness of other critical signs was limited. Maternal education, access to media, and institutional delivery were identified as key determinants of knowledge. Therefore, designing enhanced and targeted health education programs, and facility-based interventions can help bridge the critical gaps.

Acknowledgements

We would like to express out heartfelt gratitude to Addis Ababa Medical University College (AAMUC), Hargeisa Campus, for their invaluable support and cooperation throughout this study. Our deepest appreciation goes to our supervisors for their continuous guidance and encouragement. We are also sincerely thankful to the data collectors and research team members for their dedication and commitment. Finally, we extend our profound gratitude to the mothers who participated in this study; without their willingness and contribution, this research would not have been possible.

Reference

1. UNFPA. Trends in Maternal Mortality 2000-2023: Estimates by WHO, UNICEF, UNFPA, World Bank Group and UNDESA/Population Division. https://www.unfpa.org/publications/trends-maternal-mortality-2000-2023#:~:text=While%20maternal%20mortality%20spiked%20during,to%20the%20care%20they%20need. Accessed March 2025.
2. Wilson center. Global Health and Gender Policy Brief: Drivers of Global Maternal Mortality. https://www.wilsoncenter.org/publication/global-health-and-gender-policy-brief-drivers-global-maternal-mortality#:~:text=These%20inequalities%20are%20profound.,classified%20as%20late%20maternal%20deaths. Accessed December 2024.
3. UNDP. Sustainable Development Goal (SDG). Goal 3: Good health and well-being. https://www.undp.org/sustainable-development-goals/good-health. Accessed March 2025.
4. Abdurashid N, Ishaq N, Ayele K, Ashenafi N. Level of Awareness on Danger Signs During Pregnancy and Associated Factors, among Pregnant Mothers, Dire Dawa Administrative Public Health Facilities, Eastern Ethiopia. Clinics Mother Child Health 15: 290. doi:10.4172/2090-7214.1000290.
5. Thaddeus S, Maine D. Too far to walk: maternal mortality in context. *Social Science & Medicine.* 1994; 38(8):1091-1110. https://doi.org/10.1016/0277-9536(94)90226-7
6. Bolanko A, Namo H, Minsamo K, Addisu N, Gebre M. Knowledge of obstetric danger signs and associated factors among pregnant women in Wolaita Sodo town, South Ethiopia: A community-based cross-sectional study. *SAGE Open Medicine*. 2021;9. doi:10.1177/20503121211001161.

7. Wassihun, B., Negese, B., Bedada, H. *et al.* Knowledge of obstetric danger signs and associated factors: a study among mothers in Shashamane town, Oromia region, Ethiopia. *Reprod Health* **17**, 4 (2020). https://doi.org/10.1186/s12978-020-0853-z.
8. Nyirenda M, Hazemba AN, Makasa M. Determinants of knowledge of obstetric danger signs among women attending antenatal care in Eastern province, Zambia: a cross-sectional study. BMC Pregnancy Childbirth. 2025 Jul 3;25(1):719. doi: 10.1186/s12884-025-07782-y. PMID: 40610952; PMCID: PMC12224364.
9. The Somaliland Health and Demographic Survey 2020 HDS; October 2022; https://somalia.unfpa.org/sites/default/files/pub-pdf/slhds2020_report_2020.pdf. Accessed March 2025.
10. Wikipedia, Somaliland. https://en.wikipedia.org/wiki/Somaliland. Accessed March 2025.
11. Wikipedia, Hargeisa. https://en.wikipedia.org/wiki/Hargeisa. Accessed March 2025.
12. Maseresha et al. Knowledge of obstetric danger signs and associated factors among pregnant women in Erer district, Somali region, Ethiopia, BMC Women's Health. 2016; 16:30.
13. JHPIEGO. *Monitoring Birth preparedness and complication readiness: Tools Indicators for Maternal and Newborn Health*. Baltimore, Maryland: JHPIEGO Maternal and Neonatal Health Program, 2004.
14. Jewaro, M., Yenus, H., Ayanaw, Y. *et al.* Knowledge of obstetric danger signs and associated factors among mothers in Bahir Dar district, northwest Ethiopia: an institution-based cross-sectional study. *Public Health Rev* **41**, 14 (2020). https://doi.org/10.1186/s40985-020-00132-7.

15. Mestawut Mihret, Hailegebriel Wondimu. Assessment of Knowledge about Obstetric Danger Signs and Associated Factors among Pregnant Women in Debre Tabor Town, Northwest Ethiopia. Wiley online Library, Journal of Pregnancy. 2023. https://doi.org/10.1155/2023/1475500.

16. Haile, S., Mekonen, L., Shiferaw, Z., Degefu, A., Getachew, N., Alemayehu, Y., & Issak, D. (2024). Knowledge about Obstetric Danger Signs and Associated Factors among Pregnant Mothers in Jigjiga City, Somali Regional State, Eastern, Ethiopia. *East African Journal of Pastoralism*, *1*(2). Retrieved from https://eajp.et/index.php/jju/article/view/37.

17. Okoror, C. E. M., & Omuemu, V. O. (2021). Knowledge of obstetric danger signs among antenatal clinic attendees in South–South Nigeria. *Health Care for Women International*, *44*(10–11), 1363–1378. https://doi.org/10.1080/07399332.2021.1941025.

18. Tamang, S.T., Dorji, T., Yoezer, S. *et al.* Knowledge and understanding of obstetric danger signs among pregnant women attending the antenatal clinic at the National Referral Hospital in Thimphu, Bhutan: a cross-sectional study. *BMC Pregnancy Childbirth* **21**, 104 (2021). https://doi.org/10.1186/s12884-021-03580-4.

19. Nkamba, D.M., Wembodinga, G., Bernard, P. *et al.* Awareness of obstetric danger signs among pregnant women in the Democratic Republic of Congo: evidence from a nationwide cross-sectional study. *BMC Women's Health* **21**, 82 (2021). https://doi.org/10.1186/s12905-021-01234-3.

20. Dedih Suandi, Pauline Williams, Sohinee Bhattacharya, Does involving male partners in antenatal care improve healthcare utilisation? Systematic review and meta-analysis of the published literature from low- and middle-income countries, *International Health*,

Volume 12, Issue 5, September 2020, Pages 484–498, https://doi.org/10.1093/inthealth/ihz073.

21. Agbor Nathan Emeh et al. Antenatal care and determinants of obstetric danger signs awareness of immediate postpartum women at Buea Regional Hospital, Cameroon. Pan African Medical Journal. 2021;38:247. [doi: 10.11604/pamj.2021.38.247.20977].
22. Imani Ramazani B-E, Mabakutuvangilanga Ntala S-D, Katuashi Ishoso D, Rothan-Tondeur M. Knowledge of Obstetric Danger Signs among Pregnant Women in the Eastern Democratic Republic of the Congo. *International Journal of Environmental Research and Public Health*. 2023; 20(8):5593. https://doi.org/10.3390/ijerph20085593.
23. Uwiringiyimana E, Manirambona E, Byiringiro S, Nsanzimana A, Uhawenayo N, Ufitinema P, et al. (2022) Pregnant women's knowledge of obstetrical danger signs: A cross-sectional survey in Kigali, Rwanda. PLOS Glob Public Health 2(11): e0001084. https://doi.org/10.1371/journal.pgph.0001084.
24. Yunitasari, E., Matos, F., Zulkarnain, H. *et al.* Pregnant woman awareness of obstetric danger signs in developing country: systematic review. *BMC Pregnancy Childbirth* **23**, 357 (2023). https://doi.org/10.1186/s12884-023-05674-7.
25. Bhavana, B M; Yogesh, M; Padhiyar, Navneet; Damor, Naresh. Obstetric danger signs in context: A mixed methods study exploring knowledge and sociocultural factors among pregnant women. Journal of Education and Health Promotion 13(1):491, December 2024. | DOI: 10.4103/jehp.jehp_315_24.
26. Wulandari RD, Laksono AD (2020) Determinants of knowledge of pregnancy danger signs in Indonesia. PLoS ONE 15(5): e0232550. https://doi.org/10.1371/journal.pone.0232550.

27. Sungwa, E. E., Mwakanyamale, A., & Magesa, R. (2023). Assessment of knowledge on the danger signs of pregnancy among pregnant women at Sinza Palestina Hospital in Ubungo Municipality, Tanzania. *Tanzania Journal of Health Research*, *24*(4), 471–482. https://doi.org/10.4314/thrb.v24i4.

28. World Health Organization. *WHO recommendations: intrapartum care for a positive childbirth experience.* Geneva: WHO; 2018. Available from: https://www.who.int/publications/i/item/9789241550215. Accessed March 2025.

 Click here to access/download;Figure;Fig 1.tif

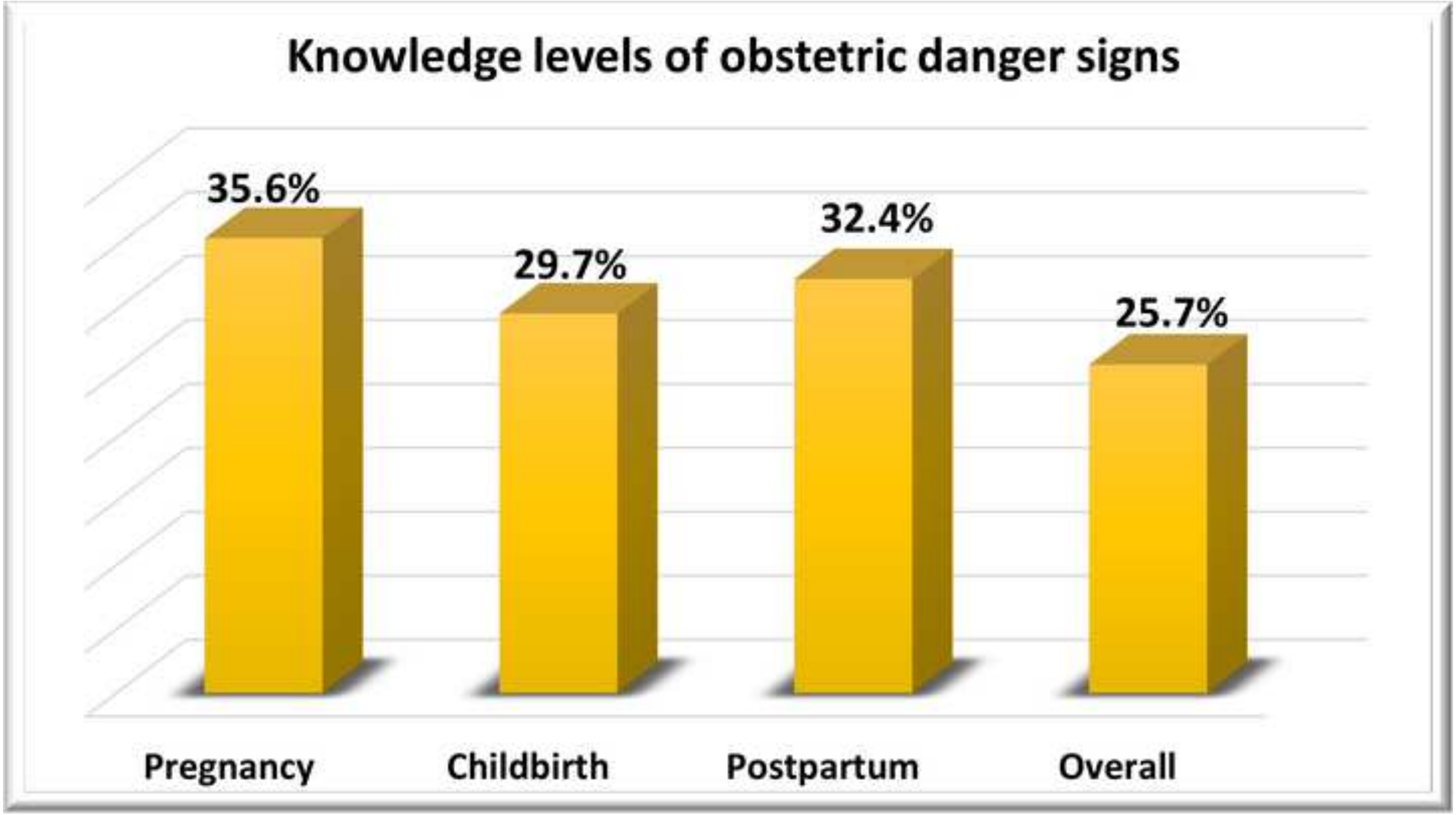

Click here to access/download
Supporting Information
S1_File_SPSS_Knowledge_ODS_PLOS.sav

Click here to access/download
Supporting Information
S2_File_xlsx_Knowledge_ODS_PLOS.xlsx

Maternal Knowledge of Obstetric Danger Signs and Related Factors among Antenatal Care Attendees in Hargeisa, Somaliland: A Cross-Sectional Analysis

Abdeta Muktar Ahmed [1*], Mohamed Abdilahi Ahmed [1], Abdulkadir Mohammed Nuh [2], Hamse Khalif Hassan [1,3]

[1] Addis Ababa Medical University College, Hargeisa C~~c~~ampus, Department of Public Health, Hargeisa, Somaliland

[2] Addis Ababa Medical University College, Hargeisa C~~c~~ampus, Department of Nutrition, Hargeisa, Somaliland

[3] International Health program, C~~c~~ollege of M~~m~~edicine, National Yang Ming Chiao Tung University (NYCU), Taipei~~ city~~, Taiwan

* Corresponding author

E-mail: abdetamuk16@gmail.com

Abstract

Background: Maternal mortality remains a major global health challenge, disproportionately affecting low- and middle-income countries. Despite international efforts, nearly 95% of maternal deaths occur in these regions, with Sub-Saharan Africa bearing the highest burden. Limited knowledge of obstetric danger signs is a key factor contributing to delays in seeking care, which in turn increases the risk of preventable maternal complications. Evidence on maternal awareness in Somaliland is scarce.

Objective: This study aimed to assess the level of knowledge of obstetric~~s~~ danger signs and associated factors among pregnant women attending antenatal care in Hargeisa, Somaliland.

Methods: A facility-based cross-sectional study was conducted from July 2 to August 4, 2022, among pregnant women attending antenatal care services. A total of 222 participants were selected using a systematic random sampling technique from eight maternal and child health centers. Data were collected through face-to-face interviews using a pretested and structured questionnaire and analyzed with SPSS version 25. Bivariate and multivariate logistic regression was carried out.

Results: About 35.6% had a good ~~were~~ knowledge ~~able~~ specifically about pregnancy related obstetric danger signs. Only 57 women (25.7%) demonstrated ~~adequate~~ overall good knowledge of obstetric danger~~s~~ signs across pregnancy, childbirth, and the postpartum periods. ~~,~~ Knowledge was highest for vaginal bleeding (65% - 67%), while less visible complications such as convulsions and blurred vision were less frequently mentioned. Multivariate analysis revealed that secondary level education or higher (AOR = 3.6, 95% CI: 1.8-7.3), access to and usage of

media (AOR = 2.6, 95% CI: 1.2-5.6), and institutional delivery (AOR = 3.4, 95% CI: 1.6-7.2), were significant predictors of knowledge.

Conclusion: Maternal knowledge of obstetric danger signs in Hargeisa was ~~alarmingly~~ low. Targeted health education, broader use of mass media, and promoting institutional delivery are critical strategies to improve and reduce preventable maternal mortality.

Key words: ~~O~~obstetric danger signs, maternal k~~k~~nowledge~~ and awareness~~, a~~a~~ntenatal care, p~~p~~regnancy complications, Hargeisa, Somaliland~~.~~

Background

~~The ~~M~~m~~aternal mortality remains a pressing global issue. In 2023, more than 700 women lost their lives to pregnancy-related and childbirth-related avoidable causes every day, or roughly one woman every two minutes [1]. Despite international efforts to address it, maternal mortality is still a serious global health concern. The startling truth is that the majority of maternal fatalities are avoidable and result from severe health disparities that occur both within and between nations and worldwide. These disparities are significant. Nearly 95% of all maternal deaths take place in low- and middle-income nations, with Sub-Saharan Africa bearing the highest risk, accounting for 70% of all maternal deaths. The 2030 United Nations Sustainable Development Goal (SDG), which aims to lower the rate of maternal fatalities to fewer than 70 per 100,000 live births, is still significantly above the current global rate of maternal deaths (223 per 100,000 live births) [2-3].

Even though, an estimated 260,000 maternal deaths, or about 700 deaths per day, occurred globally in 2023, maternal mortality has decreased by 40% since 2000, which is indicative of ongoing international efforts to provide access to basic healthcare services. No region was designated as having a very high maternal mortality ratio (MMR), and no country was predicted to have an exceptionally high MMR for the first time [14].

One of the main causes of maternal mortality in the underdeveloped world is women's, families', and birth attendants' lack of awareness about the obstetric warning signs [4]. This challenge can be better understood within the framework of the Three Delays Model, which highlights delays in (a) deciding to seek care, (b) reaching a health facility, and (c) receiving appropriate care upon arrival [5]. In this context, inadequate knowledge of obstetric danger signs primarily contributes

to the first delay, as women and their families may fail to recognize life-threatening complications and seek timely medical attention. These deaths can be decreased by educating women about the warning signs of obstetrics dangers, encouraging them to seek medical attention when necessary, and counselling them on these obstetric danger signsand providing them on counselling on these signs of obstetrics danger, as well as the right management of these complications [45]. Women should be aware of the warning signs of obstetric dangers, during pregnancy, birth, and the postpartum period, as every pregnancy faces risk. In the end, the information will enable them and their families to decide quickly to seek care from trained birth attendants [6].

Obstetric danger signs are warning indications that could point to potentially life-threatening conditions during pregnancy, labor, or the postpartum phases. Approximately bout 75% of all maternal deaths during these three phases are caused by obstetric danger signs, which include vaginal bleeding, blurred vision, lack of fetal movement, a gush of fluid from the vagina, high fever, retained placenta, foul-smelling discharge, convulsions, and severe headaches. Because mothers and family members may quickly recognize these signs without need for a complex professional diagnosis, raising maternal knowledge is a crucial approach in minimizing care-seeking delays [67]. Pregnant women who notice warning symptoms and seek prompt obstetric care could reduce maternal mortality from complications connected to childbirth in low-income countries. One of the most frequent reasons why people fail to detect complications when they arise and put off seeking care is a lack of warning signs [78]. Poor maternal and newborn outcomes result from pregnant women’s lack of awareness of obstetrics danger risk signs, which is a major cause in delays in seeking care. Designing focused treatments that raise awareness, encourage prompt care-seeking behavior, and improve birth preparedness requires an

understanding of the factors influencing pregnant women's knowledge of obstetrics danger signs [89].

According to the 2020 Somaliland Health and Demographic Survey (SLHDS), the country experiences an estimated 396 maternal deaths for every 100,000 live births. Furthermore, only 47% of pregnant women reported attending at least one antenatal care (ANC) visit, and merely 33% delivered with the assistance of skilled birth attendant [910]. Although Somaliland's maternal mortality ratio has shown a declining trend and is not among the highest in the region, it still remains far from achieving the SDG target of reducing maternal mortality to 70 per 100,000 live births by 2030.

To date, no studies have been conducted in Hargeisa, Somaliland, to assess the level of knowledge regarding obstetric danger signs and their associated factors. Understanding this knowledge gap is crucial, as improving awareness of obstetric danger signs can inform strategies to enhance maternal and health outcomes and reduce maternal mortality. Therefore, the present study aimeds to evaluate the level of knowledge of obstetric danger signs and to identify the associated factors among pregnant women attending antenatal care (ANC) services in selected health institutions in Hargeisa, Somaliland.

Materials and Methods

Study design and area

A facility-based cross-sectional study was conducted in Hargeisa, the capital of Somaliland, from July 2 to August 4, 2022. Somaliland, a self-declared autonomous state in the horn of Africa that unilaterally separated from Somalia in 1991, had an estimated population of 6.2 million [101]. Hargeisa, its capital, is home to approximately 1.2 million people [112].

Study population

The source population consisted of pregnant women attending ANC services at health institutions in Hargeisa during the study period.

Inclusion criteria: All pregnant women attending antenatal care services in the selected health institutions during the study period who were physically and mentally able and willing to participate.

Formatted: Font: Bold

~~were includ~~**Exclusion criteria**: Pregnant ~~ed~~women who ~~, whereas those who~~ were critically ill at the time of data collection, defined as those experiencing severe medical or obstetric conditions that impaired their ability to communicate or safely participate in an interview. ~~were excluded.~~

The sample size was calculated using a single population proportion formula:

$n = (Z^2 \times p \times (1-p)) / d^2$

Where Z = 1.96 (95% confidence level), p = 0.155 (~~taking into account of a~~ 15.5% ~~[13]~~ prevalence of knowledge of obstetric~~s~~ danger signs from a previous study [12]), and 5% margin of error.

$n = (1.96^2 \times 0.155 \times 0.845) / (0.05^2) \approx 201$

After adding ~~, 95% confidence level, and~~ a 10% ~~allowance for~~ non-response rate, t~~. T~~he final sample size became ~~was~~ 222.

Sampling procedure

Eight centers were selected through simple random sampling from the total of 17 Maternal and Child Health (MCH) centers to ensure representativeness and minimize selection bias.

The selected health facilities were public ~~Out of the 17~~ Maternal and Child Health (MCH) centers that provide comprehensive primary healthcare services, including antenatal care, delivery services, postnatal care, and child health services. In addition, routine maternal services including health education, nutritional counselling, basic laboratory investigations, and clinical examinations are offered. The facilities are staffed by trained healthcare professionals, including midwives and nurses. As government-run institutions, they follow standardized service delivery

Formatted: Font: Bold

Formatted: Superscript

Formatted: Superscript

Formatted: Superscript

Formatted: Superscript

Formatted: Font: 16 pt, Bold

protocols, ensuring relatively uniform care across facilities. These MCH centers are widely distributed throughout Hargeisa city, making them highly accessible and commonly utilized by the population.

~~in Hargeisa, eight were selected through simple random sampling.~~ Proportional allocation was applied to distribute the study units across the selected facilities, based on the number of ANC attendees recorded in the month preceding the survey. The sampling interval (K) was calculated as about three, meaning every third eligible woman attending ANC was invited to participate until the allocated quota for each facility was reached.

~~Variables~~ Research Instrument and measurement

Data were collected using pretested, structured, interviewer-administered questionnaire adapted from the Maternal and Newborn Health Program of JHPIEGO, an ~~d~~ affiliate of Johns Hopkins University, and other related articles [13~~4~~-14~~5~~]. The tool comprised four sections: (1) Sociodemographic characteristics, (2) Reproductive and obstetric~~s~~ history, (3) Service utilization, and (4) Knowledge of obstetric~~s~~ danger signs. The questionnaire was initially prepared in English, translated into Somali, and subsequently back-translated into English to ensure consistency and accuracy. ~~Necessary revisions were made based on feedback from the pretest.~~

Women's knowledge of obstetric danger signs was assessed by asking participants to spontaneously mention danger signs that may occur during pregnancy, childbirth, and the postpartum period, which were categorized into three groups. Knowledge of pregnancy danger signs (the study's dependent variable) was defined as correctly identifying at least three key signs, including vaginal bleeding, swollen hands/face, and blurred vision. Knowledge of childbirth/labor danger signs was defined as mentioning at least three major signs, namely

vaginal bleeding, prolonged labor, convulsions, or retained placenta. For the postpartum period, women were regarded as having good knowledge~~able~~ if they identified at least three major signs, including vaginal bleeding after delivery, loss of consciousness, and postpartum fever. Overall, a woman was considered as having good knowledge~~able~~ on obstetric~~s~~ danger signs if she was able to mention at least three key signs across each of the three phases, without prompting, in line with the JHPIEGO maternal health framework [13, 14~~5~~]. Those who mention fewer than three key danger signs were categorized as having poor knowledge. Hence, f~~F~~or analysis, the dependent variables were dichotomized into “~~**k**~~**Good knowledg~~eable~~**” and “~~**n**~~**Poor** ~~**ot**~~ **knowledg~~eable~~**.” This classification approach has been widely used in similar studies assessing maternal knowledge of obstetric danger signs in low- and middle-income settings. ~~For analysis, the dependent variables were dichotomized into “**knowledgeable**” and “**not knowledgeable**.”~~

Formatted: Font: Not Bold

Data collection procedures

Data were collected through face-to-face interview conducted by six trained BSc Nursing students. Two experienced supervisors oversaw the process. Prior to data collection, the questionnaire was pretested on 22 pregnant women at an MCH center outside the study area. Based on pretest findings, adjustments were made to improve clarity and accuracy. Training was provided to data collectors and supervisors on the objectives of the study, the data collection tool, ethical considerations, and procedures to ensure confidentiality.

Data processing and analysis

Completed questionnaires were checked daily for completeness and consistency. Data were cleaned, coded, and entered into SPSS version 25 for analysis. Descriptive statistics were used to summarize the data. Bivariate logistic regression was first performed, and variables with a p-

value < 0.2 were considered candidates for multivariate analysis. Subsequently, multivariate logistic regression was carried out to examine associations between independent variables and the outcome variable while controlling for potential confounding factors. Adjusted odds ratios (AOR) with 95% confidence interval (CI) were computed to determine the strength of associations. Statistical significance was declared at $p<0.05$.

Ethical considerations

Ethical approval for the study was obtained from the Research Ethics Committee of Addis Ababa Medical University College, Hargeisa campus (Ref. No: AAMUCHC/REC/87/22). The purpose of the study was explained to all participants, and written informed consent was obtained prior to data collection. Participation was voluntary, and respondents were assured that their information would be used solely for research purpose and kept confidential. Names and personal identifiers were not recorded to preserve anonymity.

Result

Socio-demographic characteristics

A total of 222 pregnant women participated in the study, yielding a response rate of 100%. The mean age of the respondents was 28.3 years (±6.5 SD), with a median age of 28.5 years, ranging from 17 to 43 years. More than half of the respondents (55.4%) were between 25 and 36 years of age. Most participants (201 (, 90.5%) were currently married. Regarding educational attainment, 75 (33.8%) had no formal education, 53 (23.9%) had completed primary education, 38 (17.1%) had secondary education, and 56 (25.2%) attained higher education. By contrast, more than half of their spouses (121 (, 54.5%) had higher education, while only 59 (26.6%) had primary or no

formal education. In terms of occupation, the majority of respondents (153 (,68.9%) were merchants, 49 (22.1%) were housewives/unemployed, and 20 (9%) were formally employed. Among spouses, 149 (67.1%) were employed, 62 (27.9%) were merchants, and 11 (5%) were unemployed. The mean monthly household income was 561 USD. A large proportion (152 (, 68.5%) reported monthly incomes of ≤500 USD. Additionally, 142 respondents (64%) had access to mainstream or social media (Table 1).

Table 1. Socio-demographic characteristics of the respondents (n=222)

**Variables**	**Category**	**Frequency (n)**	**Percentage (%)**
Age (years)	≤24	70	31.5
	25-36	123	55.4
	≥37	29	13.1
Marital status	Currently married	201	90.5
	Widowed/Divorced	21	9.5
Educational status (women)	No formal education	75	33.8
	Primary	53	23.9
	Secondary	38	17.1
	Higher education	56	25.2
Educational status (spouse)	No formal education	36	16.2
	Primary	23	10.4
	Secondary	42	18.9
	Higher education	121	54.5
Occupation (women)	Merchant	153	68.9
	Housewife/Unemployed	49	22.1
	Employed	20	9
Occupation (Spouse)	Employed	149	67.1
	Merchant	62	27.9
	Unemployed	11	5

Monthly family income (USD)	≤500	152	68.5
	501-1000	30	13.5
	>1000	40	18
Access to media	Yes	142	64
	No	80	36

Obstetric~~s~~ characteristics

The age at first marriage ranged from 14 to 28 years, with a mean of 18.9 years. Half of the respondents ~~(~~111 (~~,~~50%) married between the age of 14-18 years. The age at first pregnancy ranged from 16 to 30 years, with a mean of 20 years; more than half ~~(~~119 (~~,~~53.6%) conceived between 19-24 years. At the time of data collection, nearly half of the respondents ~~(~~102 (~~,~~45.9%) were in their third trimester.

Number of pregnancies ~~Parity~~ ranged from 1 to 11 ~~pregnancies~~, with a mean of 4.5, of which ~~.~~ ~~Among them,~~ 95 (42.8%) had experienced 4-6 pregnancies. Meanwhile, ~~, and~~ 136 participants (61.3%) reported having up to three births. Regarding pregnancy intention, 144 (64.9%) stated ~~reported~~ their current pregnancy was planned, while 78 (35.1%) ~~stated~~ described otherwise. The~~A~~ majority of ~~the~~ participants, ~~(~~178 (~~,~~80.2%) reported not experiencing any obstetric~~s~~ danger signs during their current pregnancy (Table 2).

Table 2. Obstetric variables of the study participants (n=222)

**Variables**	**Category**	**Frequency (n)**	**Percent (%)**
Age at first marriage (years)	14-18	111	50
	19-23~~4~~	9~~8~~2	41.4~~.1~~

	≥245	193	85.69
Age at first pregnancy (years)	16-198	1080	48.36
	2019-243	84119	537.68
	≥245	230	130.54
Trimester (Current pregnancy)	First	54	24.5
	Second	66	29.7
	Third	102	45.9
Number of pregnancies	≤3	83	37.4
	4-6	95	42.8
	>6	44	19.8
Number of births	≤3	136	61.3
	4-6	62	27.9
	>6	24	10.8
Current pregnancy intended	Yes	144	64.9
	No	78	35.1
History of danger signs	Yes	44	19.8
	No	178	80.2

Maternal Health Service Utilization

With respect to ANC visits during the current pregnancy, 63 women (28.4%) had attended only once, while 66 (29.7%), 55 (24.8%), and 38 (17.2%) had attended two, three, and four or more visits, respectively. More than two-thirds (158 (, 71.2%) had received health education about obstetric danger signs during ANC visits, whereas 64 (28.8%) had not.

Among 209 respondents with previous childbirth experience, 128 (61.2%) delivered their last baby in a health institution, while 81 (38.8%) delivered at home. The majority (187 (, 89.5%) reported spontaneous vaginal delivery, followed by 15 (7.2%) instrumental delivery, and 7 (3.315, 7.2%) and cesarean section (7, 3.3%).

The reported travel time to the nearest health facility ranged from 1 to 120 minutes, with a mean of 33 minutes. More than half, (130 (,58.6%) lived within a 30-minutes walking distance. Regarding decision-making autonomy, 116 (52.3%) reported making decisions independently, 82 (36.9%) jointly with their partner, and 24 (10.8%) reported that decisions were solely by their partner. Only 85 (38.3%) discussed maternal health service utilization with their spouses, while the majority, (137 (,61.7%) did not (Table 3).

Table 3. Maternal health service utilization (n=222)

**Variables**	**Category**	**Frequency (n)**	**Percentage (%)**
ANC visits (current pregnancy)	1-3	184	82.9
	≥4	38	17.2
Received health education on danger signs	Yes	158	71.2
	No	64	28.8
Place of last delivery (n=209)	Home	81	38.8
	Health institution	128	61.2
Mode of delivery (n=209)	Spontaneous vaginal delivery (SVD)	187	89.5
	Instrumental Delivery	15	7.2
	Cesarean section	7	3.3
Time to nearest health facility (walk)	≤30 min	130	58.6
	>30 min	92	41.4
Decision making power	Self	116	52.3
	Joint (both)	82	36.9
	Partner only	24	10.8
Discussion with spouse on maternal services	Yes	85	38.3
	No	135	61.7

Knowledge of obstetric Danger Signs

Respondents were asked to list key obstetric~~s~~ danger signs spontaneously. During pregnancy, the most commonly mentioned signs were vaginal bleeding by ~~(~~144 participants (~~,~~64.9%), blurred vision ~~(~~126 (~~,~~56.8%), swollen face and limbs ~~(~~119 (~~,~~53.6%), severe headache ~~(~~108 (~~,~~48.6%), hypertension ~~(~~75 (~~,~~33.8%), and convulsion~~s~~ ~~(~~73 (~~,~~32.9%).

During childbirth, prolonged labor by ~~(~~150 respondents (~~,~~67.6%) and vaginal bleeding by ~~(~~149 (~~,~~67.1%) were the most frequently mentioned danger signs, followed by retained placenta ~~(~~115 (~~,~~51.8%), hypertension ~~(~~96 (~~,~~43.2%), and convulsion~~s~~ ~~(~~92 (~~,~~41.4%).

In the postnatal period, vaginal bleeding ~~(~~145 (~~,~~65.3%) was the most frequently mentioned sign, followed by high fever ~~(~~131 (~~,~~59%), foul-smelling vaginal discharge ~~(~~98 (~~,~~44.1%), severe weakness ~~(~~93 (~~,~~41.9%), and difficulty breathing ~~(~~83 (~~,~~37.4%) (Table 4).

Table 4. Knowledge of obstetric danger signs mentioned by the respondents (n=222)

(a) During pregnancy

Danger sign	Frequency (n)	Percent (%)
Vaginal bleeding	144	64.9
Blurred vision	126	56.8
Swollen face/limbs	119	53.6
Severe headache	108	48.6
Hypertension	75	33.8
Convulsions	73	32.9
Persistent nausea /vomiting	71	32
Malpresentation/position	71	32
Reduced/absent fetal movement	68	30.6
Severe abdominal cramp	66	29.7
Leakage of amniotic fluid	46	20.7

(b) During childbirth

Danger sign	Frequency (n)	Percent (%)
Prolonged labor	150	67.6
Vaginal bleeding	149	67.1
Retained placenta	115	51.8
Hypertension	96	43.2
Convulsions	92	41.4
Cessation of labor pain	81	36.5
High fever	80	36
Swollen face/limbs	74	33.3
Severe headache	71	32
Malpresentation/position	59	26.6
Blurred vision	52	23.4
Severe abdominal cramps	46	20.7

(c) During postnatal period

Danger sign	Frequency (n)	Percent (%)
Vaginal bleeding	145	65.3
High fever	131	59
Foul-smelling vaginal discharge	98	44.1
Severe weakness	93	41.9
Difficulty breathing	83	37.4
Swollen face/limbs	73	32.9
Severe headache	61	27.6
Unconsciousness	58	26.1
Uterine prolapse	50	22.5
Blurred vision	48	21.6
Convulsions	45	20.3

Overall, only 79 respondents (35.6%) demonstrated adequate knowledge of obstetric~~s~~ danger signs during pregnancy. ~~, which was the study's dependent variable.~~ Knowledge of danger signs was lower during childbirth, with 66 respondents (29.7%) identified as having good knowledge~~able~~, and during the postnatal period, with 72 respondents (32.4%). When considering knowledge across all the three periods, only 57 respondents (25.7%) demonstrated adequate overall knowledge of obstetric danger signs (Figure 1).~~.~~ Notably, 35 participants (15.8%) were unable to mention even one of the three key danger signs across all three phases. ~~(Figure 1).~~

Figure 1. Knowledge of obstetric danger signs during pregnancy, childbirth, and postpartum periods (n=222)

Factors Associated with Knowledge of Obstetric Danger Signs

Bivariate and multivariate logistic regression analyses were conducted to identify factors associated with knowledge of key obstetric~~s~~ danger signs during pregnancy. In the bivariate analysis, women's educational status, access to media, place of delivery, decisions-making autonomy, and receipt of health education during ANC were significantly associated. In the multivariate analysis, three factors remained significant predictors: women's education level, access to media, and place of delivery.

Women with secondary education or higher were 3.6 times more likely to have good ~~be~~ knowledge~~able~~ compared to those with primary education or less (AOR = 3.6, 95% CI: 1.8-7.3, $p < 0.001$). Access to mainstream or social media was also significant; women with such access were 2.6 times more likely to have good ~~be~~ knowledge~~able~~ (AOR = 2.6, 95% CI: 1.2-5.6, $p = 0.016$). Similarly, women who delivered their last child in a health facility were 3.4 times more likely to have good ~~be~~ knowledge~~able~~ compared to those who delivered at home (AOR = 3.4, 95% CI: 1.6-7.2, $p = 0.001$) (Table 5).

Table 5. Factors associated with knowledge of obstetric danger signs (n=222)

**Variable**	**Category**	**Good k~~K~~nowledge~~able~~ n (%)**	**Poor ~~Not~~ knowledge~~able~~ n (%)**	**COR (95% CI)**	**AOR (95% CI)**
Education	Primary or less	25 (31.6%)	103 (72%)	1	1

	Secondary or more	54 (68.4%)	40 (28%)	5.5 (3.0-10.1) p<0.001	3.6 (1.8-7.3) p<0.001
Access to media	Yes	66 (83.5%)	76 (53.1)	4.5 (2.3-8.82) p<0.001	2.6 (1.2-5.6) p=0.016
	No	13 (16.5%)	67 (46.9%)	1	1
Place of last delivery	Home	13 (17.1%)	68 (51.1%)	1	1
	Hospital	63 (82.9%)	65 (48.9%)	5.0 (2.5- 10) p<0.001	3.4 (1.6-7.2) p<0.001

Discussion

This study assessed maternal knowledge of obstetrics danger signs among women attending antenatal care in Hargeisa, Somaliland, and identified key socio-demographic and service-related predictors.

The findings revealed that knowledge of obstetric danger signs during pregnancy was low, with only slightly more than one-third of women (35.6%) classified as having good knowledge~~able~~. This indicates a significant proportion of women may lack necessary awareness to recognize early warning signs of complications, potentially delaying timely health-seeking behavior and increasing the risk of adverse maternal outcomes. The relatively low knowledge observed in this study may be attributed to limited health education coverage, cultural norms influencing communication about reproductive health, and gaps in community-based health promotion. In line with this finding, ~~This result aligns with~~ studies conducted in Debre Tabor, Ethiopia (39%) [15~~6~~]; Jigjiga, Ethiopia (37.9%) [16~~6~~]; and Zambia (35%) [8~~9~~] also reported comparable ~~, where awareness~~ insufficient awareness of obstetric danger signs ~~remained insufficient~~ despite the implementation of maternal health programs. Similarly, lower levels of knowledge were reported in Dire Dawa, Ethiopia (24.1%) [4~~5~~] and Southern Nigeria (24.9%) [17~~7~~]. ~~The relatively low knowledge observed in this study may be attributed to limited health education coverage, cultural norms influencing communication about reproductive health, and gaps in community-based health promotion.~~

The findings revealed considerable ~~substantial~~ gaps in awareness, particularly concerning pregnancy-related complications. While a proportion of respondents were able to identify one or more key danger signs, ~~alarmingly,~~ a considerable number of women were unable to mention any, indicating a critical gap in basic knowledge. ~~.~~This gap in knowledge raises serious public health concerns, as unawareness of warning symptoms may delay timely health-seeking behavior and increase the risk of adverse maternal outcomes. Additionally, these women would be at higher risk of failing to seek emergency care promptly if complications arise. Similar patterns have been documented in resource-limited settings, such as in Bhutan [18~~8~~] and in a nationwide

study conducted in the Democratic Republic of Congo (DRC) [19~~9~~], where women often rely on traditional beliefs, family influence, or wait until complic~~l~~ations worsen before deciding to seek care. The lack of ~~K~~knowledge among these women reflects missed opportunities for health education during routine antenatal visits, which are intended to serve not only as check-ups but also as platforms for prevention counselling.

Despite moderate ANC utilization, the number of women achieving the recommended four visits was optimal. Furthermore, in our study, spousal communication regarding maternal health was notably low (38.3%), a factor that may hinder timely decision-making during emergencies, in contrast to male involvement promoting positive maternal health service utilization. A systematic review and meta-analysis from low- and middle-income countries likewise positively identified male involvement as a positive factor to maternal health-seeking behavior including knowledge of obstetric danger signs~~.~~ [20~~20~~].

Vaginal bleeding emerged as the most commonly recognized danger sign across pregnancy, childbirth, and the postpartum period, suggesting that more visible symptoms are more easily recognized. In contrast, danger signs such as convulsion and blurred vision were less commonly mentioned, highlighting knowledge gaps in recognizing less visible but equally life-threatening complications like pre-eclampsia and eclampsia.

~~.~~This finding aligns with studies in Shashamene, Ethiopia [7~~8~~], Cameroon [21~~1~~], eastern DRC [22~~2~~], and Rwanda [23~~3~~], as well as with a systematic review from developing countries [24~~4~~], where bleeding was consistently the most frequently recognized danger sign~~, likely due to its obvious and visible nature~~. ~~In contrast, danger signs such as convulsion and blurred vision were less commonly mentioned, highlighting knowledge gaps in recognizing less visible but equally life-threatening complications like pre-eclampsia and eclampsia.~~

Educational status appeared to play an important role in knowledge levels, as women with secondary or higher education were more likely to correctly identify danger signs than their less-educated counterparts with little or no schooling. This reflects the influence of education on awareness levels, ~~This ~~underscoring ~~es ~~the critical role of female education in enhancing maternal health outcomes.~~–~~ This finding aligns with evidence from other studies conducted in Bahir Dar, Ethiopia [14~~5~~], Cameroon [21~~1~~], India [25~~5~~], Indonesia [26~~6~~], southern Nigeria [17~~7~~], and Tanzania [27~~7~~], where literacy was positively associated with maternal knowledge and health seeking behavior. ~~This underscores the critical role of female education in enhancing maternal health outcomes.~~

Access to and use of media is one of factors associated with good danger signs knowledge, ~~also played a crucial role, ~~suggesting that health promotion through radio, television, and social media platforms can play a crucial role in ~~-substantially ~~improving~~e~~ maternal awareness. -These results highlight the critical role of accessible information channels in shaping maternal health outcomes, particularly in settings where formal educations levels may be relatively low. Such media exposures and campaigns not only increased awareness of obstetric danger signs but also encouraged greater utilization of community-based interventions to bridge knowledge gaps and promote positive health-seeking behavior. ~~These results highlight the critical role of accessible information channels in shaping maternal health outcomes, particularly in settings where formal educations levels may be relatively low. ~~This finding is consistent with studies in Jigjiga, Ethiopia [16~~6~~], and Indonesia [26~~6~~], where mass media health education was proven~~d~~ effective in enhancing health literacy among women of reproductive age. ~~Such media exposures and campaigns not only increased awareness of obstetric danger signs but also encouraged greater utilization of community-based interventions to bridge knowledge gaps and promote positive~~

health-seeking behavior. These results highlight the critical role of accessible information channels in shaping maternal health outcomes, particularly in settings where formal educations levels may be relatively low.

Institutional delivery was another strong predictor of knowledge, as women who delivered in health facilities were more likely to recall danger signs, possibly due to counselling received during delivery and postnatal care. This suggests that exposure to skilled care environments enhanced maternal awareness through repeated counselling opportunities. Moreover, it underlines the role of health facilities as important platforms for reinforcing health education beyond routine antenatal services. This finding is consistent with supports WHO recommendations emphasizing emphasizing skilled birth attendance as a key avenue for maternal health education [28]. Similarly,Furthermore, evidence from a systematic review in developing counties [244], and a study conducted in Zambia [89] support this association, stressing highlighting the role of facility-based care in improving women's awareness.

Overall, the results suggest that while some progress has been made, substantial gaps remain in maternal knowledge, particularly concerning less obvious danger signs and across the pregnancy, childbirth and postnatal periods.

Strengths and limitations

The study provides context-specific evidence on maternal knowledge of obstetric danger signs, which is essential for designing targeted interventions. In addition, the use of a structured

questionnaire allowed for systematic assessment of knowledge across multiple domains. However, the following limitations should be considered. The cross-sectional design precludes establishing cause-and-effect relationships. Self-reported knowledge may be subject to recall or social desirability bias. Furthermore, the study was limited to women attending health facilities, which may not represent those who do not access ANC services and who might have even lower awareness. Despite its limitations, the study provides valuable evidence for public health by identifying gaps in maternal knowledge of obstetric danger signs.

Conclusion

This study demonstrated that knowledge of obstetric danger signs among pregnant women in Hargeisa is ~~alarmingly~~ low, with only about one-third of the respondents having good knowledge~~able~~ about danger signs during pregnancy and barely one-quarter demonstrating overall adequate knowledge. It highlights critical gaps in maternal knowledge, with a particular concerning proportion of women unable to identify any key warning signs. Vaginal bleeding was the most commonly recognized danger sign, but awareness of other critical signs was limited. Maternal education, access to media, and institutional delivery were identified as key determinants of knowledge. Therefore, designing enhanced and targeted health education programs, and facility-based interventions can help bridge the critical gaps.

Acknowledgements

We would like to express out heartfelt gratitude to Addis Ababa Medical University College (AAMUC), Hargeisa Campus, for their invaluable support and cooperation throughout this study. Our deepest appreciation goes to our supervisors for their continuous guidance and encouragement. We are also sincerely thankful to the data collectors and research team members for their dedication and commitment. Finally, we extend our profound gratitude to the mothers who participated in this study; without their willingness and contribution, this research would not have been possible.

Reference

1. UNFPA. Trends in Maternal Mortality 2000-2023: Estimates by WHO, UNICEF, UNFPA, World Bank Group and UNDESA/Population Division. https://www.unfpa.org/publications/trends-maternal-mortality-2000-2023#:~:text=While%20maternal%20mortality%20spiked%20during,to%20the%20care%20they%20need. Accessed on March 2025.

1. World Health Organization. Trends in maternal mortality 2000 to 2023: estimates by WHO, UNICEF, UNFPA, World Bank Group and UNDESA/Population Division. https://www.who.int/publications/i/item/9789240108462. Accessed on April 2025.

2. Wilson center. Global Health and Gender Policy Brief: Drivers of Global Maternal Mortality. https://www.wilsoncenter.org/publication/global-health-and-gender-policy-brief-drivers-global-maternal-mortality#:~:text=These%20inequalities%20are%20profound.,classified%20as%20late%20maternal%20deaths. Accessed on December 2024.

3. UNDP. Sustainable Development Goal (SDG). Goal 3: Good health and well-being. https://www.undp.org/sustainable-development-goals/good-health. Accessed on March 2025.

4.1. UNFPA. Trends in Maternal Mortality 2000-2023: Estimates by WHO, UNICEF, UNFPA, World Bank Group and UNDESA/Population Division. https://www.unfpa.org/publications/trends-maternal-mortality-2000-2023#:~:text=While%20maternal%20mortality%20spiked%20during,to%20the%20care%20they%20need. Accessed on March 2025.

5.4. Abdurashid N, Ishaq N, Ayele K, Ashenafi N. Level of Awareness on Danger Signs During Pregnancy and Associated Factors, among Pregnant Mothers, Dire Dawa

Administrative Public Health Facilities, Eastern Ethiopia. Clinics Mother Child Health 15: 290. doi:10.4172/2090-7214.1000290.

5. Thaddeus S, Maine D. Too far to walk: maternal mortality in context. *Social Science & Medicine*. 1994; 38(8):1091-1110. https://doi.org/10.1016/0277-9536(94)90226-7

~~6.~~1.~~Mestawut Mihret, Hailegebriel Wondimu. Assessment of Knowledge about Obstetric Danger Signs and Associated Factors among Pregnant Women in Debre Tabor Town, Northwest Ethiopia. Wiley online Library, Journal of Pregnancy. 2023. https://doi.org/10.1155/2023/1475500.~~

~~7.~~6. Bolanko A, Namo H, Minsamo K, Addisu N, Gebre M. Knowledge of obstetric danger signs and associated factors among pregnant women in Wolaita Sodo town, South Ethiopia: A community-based cross-sectional study. *SAGE Open Medicine*. 2021;9. doi:10.1177/20503121211001161.

~~8.~~7. Wassihun, B., Negese, B., Bedada, H. *et al.* Knowledge of obstetric danger signs and associated factors: a study among mothers in Shashamane town, Oromia region, Ethiopia. *Reprod Health* **17**, 4 (2020). https://doi.org/10.1186/s12978-020-0853-z.

~~9.~~8. Nyirenda M, Hazemba AN, Makasa M. Determinants of knowledge of obstetric danger signs among women attending antenatal care in Eastern province, Zambia: a cross-sectional study. BMC Pregnancy Childbirth. 2025 Jul 3;25(1):719. doi: 10.1186/s12884-025-07782-y. PMID: 40610952; PMCID: PMC12224364.

~~10.~~9. The Somaliland Health and Demographic Survey 2020 HDS; October 2022; https://somalia.unfpa.org/sites/default/files/pub-pdf/slhds2020_report_2020.pdf~~.Accessed.~~ ~~on M~~Accessed March 2025.

Formatted: Font: Italic

Field Code Changed

~~11.~~10. Wikipedia, Somaliland. https://en.wikipedia.org/wiki/Somaliland. Accessed ~~on~~ March 2025.

~~12.~~11. Wikipedia, Hargeisa. https://en.wikipedia.org/wiki/Hargeisa. Accessed ~~on~~ March 2025.

~~13.~~12. Maseresha et al. Knowledge of obstetric danger signs and associated factors among pregnant women in Erer district, Somali region, Ethiopia, BMC Women's Health. 2016; 16:30.

~~14.~~13. JHPIEGO. *Monitoring Birth preparedness and complication readiness: Tools Indicators for Maternal and Newborn Health*. Baltimore, Maryland: JHPIEGO Maternal and Neonatal Health Program, 2004.

~~15.~~14. Jewaro, M., Yenus, H., Ayanaw, Y. *et al.* Knowledge of obstetric danger signs and associated factors among mothers in Bahir Dar district, northwest Ethiopia: an institution-based cross-sectional study. *Public Health Rev* **41**, 14 (2020). https://doi.org/10.1186/s40985-020-00132-7.

15. Mestawut Mihret, Hailegebriel Wondimu. Assessment of Knowledge about Obstetric Danger Signs and Associated Factors among Pregnant Women in Debre Tabor Town, Northwest Ethiopia. Wiley online Library, Journal of Pregnancy. 2023. https://doi.org/10.1155/2023/1475500.

16. Haile, S., Mekonen, L., Shiferaw, Z., Degefu, A., Getachew, N., Alemayehu, Y., & Issak, D. (2024). Knowledge about Obstetric Danger Signs and Associated Factors among Pregnant Mothers in Jigjiga City, Somali Regional State, Eastern, Ethiopia. *East African Journal of Pastoralism*, *1*(2). Retrieved from https://eajp.et/index.php/jju/article/view/37.

17. Okoror, C. E. M., & Omuemu, V. O. (2021). Knowledge of obstetric danger signs among antenatal clinic attendees in South–South Nigeria. *Health Care for Women International*, *44*(10–11), 1363–1378. https://doi.org/10.1080/07399332.2021.1941025.
18. Tamang, S.T., Dorji, T., Yoezer, S. *et al.* Knowledge and understanding of obstetric danger signs among pregnant women attending the antenatal clinic at the National Referral Hospital in Thimphu, Bhutan: a cross-sectional study. *BMC Pregnancy Childbirth* **21**, 104 (2021). https://doi.org/10.1186/s12884-021-03580-4.
19. Nkamba, D.M., Wembodinga, G., Bernard, P. *et al.* Awareness of obstetric danger signs among pregnant women in the Democratic Republic of Congo: evidence from a nationwide cross-sectional study. *BMC Women's Health* **21**, 82 (2021). https://doi.org/10.1186/s12905-021-01234-3.
20. Dedih Suandi, Pauline Williams, Sohinee Bhattacharya, Does involving male partners in antenatal care improve healthcare utilisation? Systematic review and meta-analysis of the published literature from low- and middle-income countries, *International Health*, Volume 12, Issue 5, September 2020, Pages 484–498, https://doi.org/10.1093/inthealth/ihz073.
21. Agbor Nathan Emeh et al. Antenatal care and determinants of obstetric danger signs awareness of immediate postpartum women at Buea Regional Hospital, Cameroon. Pan African Medical Journal. 2021;38:247. [doi: 10.11604/pamj.2021.38.247.20977].
22. Imani Ramazani B-E, Mabakutuvangilanga Ntala S-D, Katuashi Ishoso D, Rothan-Tondeur M. Knowledge of Obstetric Danger Signs among Pregnant Women in the Eastern Democratic Republic of the Congo. *International Journal of Environmental Research and Public Health*. 2023; 20(8):5593. https://doi.org/10.3390/ijerph20085593.

23. Uwiringiyimana E, Manirambona E, Byiringiro S, Nsanzimana A, Uhawenayo N, Ufitinema P, et al. (2022) Pregnant women's knowledge of obstetrical danger signs: A cross-sectional survey in Kigali, Rwanda. PLOS Glob Public Health 2(11): e0001084. https://doi.org/10.1371/journal.pgph.0001084.

24. Yunitasari, E., Matos, F., Zulkarnain, H. *et al.* Pregnant woman awareness of obstetric danger signs in developing country: systematic review. *BMC Pregnancy Childbirth* **23**, 357 (2023). https://doi.org/10.1186/s12884-023-05674-7.

25. Bhavana, B M; Yogesh, M; Padhiyar, Navneet; Damor, Naresh. Obstetric danger signs in context: A mixed methods study exploring knowledge and sociocultural factors among pregnant women. Journal of Education and Health Promotion 13(1):491, December 2024. | DOI: 10.4103/jehp.jehp_315_24.

26. Wulandari RD, Laksono AD (2020) Determinants of knowledge of pregnancy danger signs in Indonesia. PLoS ONE 15(5): e0232550. https://doi.org/10.1371/journal.pone.0232550.

~~27.~~ Sungwa, E. E., Mwakanyamale, A., & Magesa, R. (2023). Assessment of knowledge on the danger signs of pregnancy among pregnant women at Sinza Palestina Hospital in Ubungo Municipality, Tanzania. *Tanzania Journal of Health Research*, *24*(4), 471–482. https://doi.org/10.4314/thrb.v24i4.

27.

World Health Organization. *WHO recommendations: intrapartum care for a positive childbirth experience.* Geneva: WHO; 2018. Available from: https://www.who.int/publications/i/item/9789241550215. Accessed March 2025.

28.

Formatted: Font: (Default) Times New Roman

Formatted: List Paragraph, Numbered + Level: 1 + Numbering Style: 1, 2, 3, … + Start at: 1 + Alignment: Left + Aligned at: 0.25" + Indent at: 0.5", Tab stops: Not at 0.06" + 2.99"

Formatted: Font: Italic

Formatted: Font: (Default) Times New Roman

Response to Reviewers

We would like to sincerely thank the Journal staffs, Editor and the Reviewer for their valuable comments and constructive feedback. We have carefully revised the manuscript accordingly and believe that the changes have significantly improved its clarity, rigor, and overall quality. All comments have been addressed point-by-point below.

Responses to Journal requirements

We have addressed all the requested issues as follows:

Response to Reviewers:
We have uploaded the "Response to Reviewers" document as a separate file under the appropriate file type, including detailed point-by-point responses to all comments.

Data Availability and Minimal Dataset:
We confirm that the minimal dataset required to reproduce the findings of this study is included in the Supporting Information files. The dataset contains all variables and data used in the analysis, including those underlying the results presented in tables and figures.

Data Format:
In addition to the original SPSS (.sav) file, we have provided the dataset in Excel (.xlsx) format to facilitate accessibility and review.

Anonymization:
We confirm that all datasets have been fully anonymized. All personal identifiers and sensitive information have been permanently removed, and no identifiable data are included in the uploaded files.

Requirement 1: PLOS ONE formatting

Response:

We have revised the manuscript to comply with PLO ONE formatting guidelines, including structure, headings, and general style. The title page and manuscript formatting have been aligned with the provided templates.

Requirement 2: Reviewer-suggested citations

We carefully evaluated suggested reference and included because it was directly relevant to our study. No irrelevant citation was added.

Requirement 3: Reference list accuracy and reattracted articles

Response:

We thoroughly viewed the reference list to ensure completeness and accuracy. We confirm that none of the cited articles are retracted. Formatting inconsistencies have been corrected, and references have been standardized according to journal requirements.

Responses to Editorial Comments

Editor Comment 1:

The third paragraph under the introduction can be summarized using the 3 delays model by Deborah Maine.

Response

Thank you for this important suggestion. We have revised the third paragraph of the Introduction to explicitly frame the discussion within the Three Delays Model, clearly explaining how inadequate knowledge of obstetric danger signs contributes primarily to delays in decision-making to seek care.

Editor Comment 2:

Under the study population, highlight the inclusion and exclusion criteria

Response

We have revised the "Study Population" section to clearly present the inclusion and exclusion criteria under separate subheadings for better clarity and readability

Editor Comment 3:

Replace "Variable and measurement" on page 8 with "Research Instrument and measurements."

Response:

This has been corrected. The section now titled **“Research Instrument and measurement”** in accordance with the recommendation.

Editor Comment 4:

Under the discussion, interpret the findings before comparison with extant literature

Response:

We have revised the Discussion section to first interpret the key findings of the study before comparing them with existing literature. This improves logical flow and strengthens the analytical depth of the discussion.

Responses to Reviewer #1

Comment 1:

Clarify the calculation of sample size

Response:

We have clarified the sample size calculation by explicitly presenting the formula, defining all parameters (Z, p, d), and showing the calculation steps. This has been revised in the methods section.

Comment 2:

Explain the criteria of selection of clinic and describe the antenatal services

Response:

We have expanded the description of the selected health facilities in the methods section. Specifically, we clarified that the selected facilities are public Maternal and Child Health (MCH) centers, described the services provided (ANC, delivery, postnatal care, health education, laboratory services), and explained that they follow standardized protocols and are staffed by

trained healthcare professionals. The rationale for selection and representativeness has also been clarified.

Comment 3:

What is the definition of good, low knowledge level? and based on which criteria or KPI?

Response:

We have clearly defined “good knowledge” and poor knowledge in the Methods section. The classification is based on the ability of respondents to spontaneously mention at least three key obstetric danger signs across pregnancy, childbirth, and postpartum periods., in line with the JHPIEGO maternal health framework and previous literature. This has now been explicitly explained.

Responses to Reviewer #2

Comment 1:

Spelling and grammatical errors need correction

Response:

We have carefully reviewed the entire manuscript and corrected all spelling and grammatical errors. The manuscript has been thoroughly edited to ensure clarity, consistency, and adherence to standard academic English.

Comment 2:

What did you consider as being “critically ill”?

Response:

Thank you for this important clarification. We have revised the manuscript to explicitly define “critically ill” participants in the Methods section. In this study, critically ill pregnant women were defined as those experiencing severe medical or obstetric conditions that impaired their ability to communicate or safely participate in an interview. This clarification has been added under the exclusion criteria.

Comment 3:

Does parity as used here refer to total pregnancies including miscarriages or to births only?

Response:

We appreciate this important observation. It was pure typographic error. It was corrected and clearly stated in the Result section.

Comment 4:

The age at first marriage range categorisation is not of uniform interval.

Response:

Thank you for highlighting this issue. We have revised the categorization of age at first marriage to ensure uniform interval grouping. The categories have been adjusted to consistent intervals to improve clarity and comparability of the data.

Comment 5:

Similarly, age at first pregnancy range intervals is not uniform.

Response:

We appreciate this observation. The categorization of age at first pregnancy has been revised to ensure consistent interval groupings across categories. This change has been made in the Result section (Table 2).

Comment 6:

Considering the limitations of the study, the word “alarmingly” should not be used.

Response:

Thank you for this valuable suggestion. We agree that the term alarmingly” may overstate the findings given the study design. The word has been removed.

Comment 7:

This reference is the same as reference number 1.

Response:

Thank you for pointing out this duplication. We have carefully reviewed the reference list and identified the repeated citation. The duplicate reference has been removed, and the reference list has been updated accordingly. All in-text citations have been checked and renumbered to ensure consistency and accuracy throughout the manuscript.

Conclusion

We believe that the manuscript has been significantly improved the Editor's and reviewers' comments. We sincerely appreciate the time and effort invested in reviewing our work and hope that the revised manuscript meets the journal's expectations.

Thank you for your consideration.

Sincerely,

Abdeta Muktar Ahmed (On behalf of all authors)
