# Supplementary material for: Maternal knowledge of obstetric danger signs and related factors among antenatal care attendees in Hargeisa, Somaliland: A cross-sectional analysis
Source: PLoS One. 2026 Jul 15;21(7):e0353778. doi: 10.1371/journal.pone.0353778 (PMC13372116; doi:10.1371/journal.pone.0353778)
Supplement: Attachment — Submitted filename: Response to Reviewers.docx [file pone.0353778.s006.docx]

**Response to Reviewers**

We would like to sincerely thank the Journal staffs, Editor and the Reviewer for their valuable comments and constructive feedback. We have carefully revised the manuscript accordingly and believe that the changes have significantly improved its clarity, rigor, and overall quality. All comments have been addressed point-by-point below.

**Responses to Journal requirements**

We have addressed all the requested issues as follows:

**Response to Reviewers:**
We have uploaded the “Response to Reviewers” document as a separate file under the appropriate file type, including detailed point-by-point responses to all comments.

**Data Availability and Minimal Dataset:**
We confirm that the minimal dataset required to reproduce the findings of this study is included in the Supporting Information files. The dataset contains all variables and data used in the analysis, including those underlying the results presented in tables and figures.

**Data Format:**
In addition to the original SPSS (.sav) file, we have provided the dataset in Excel (.xlsx) format to facilitate accessibility and review.

**Anonymization:**
We confirm that all datasets have been fully anonymized. All personal identifiers and sensitive information have been permanently removed, and no identifiable data are included in the uploaded files.

**Requirement 1: PLOS ONE formatting**

**Response:**

We have revised the manuscript to comply with PLO ONE formatting guidelines, including structure, headings, and general style. The title page and manuscript formatting have been aligned with the provided templates.

**Requirement 2: Reviewer-suggested citations**

We carefully evaluated suggested reference and included because it was directly relevant to our study. No irrelevant citation was added.

**Requirement 3: Reference list accuracy and reattracted articles**

**Response:**

We thoroughly viewed the reference list to ensure completeness and accuracy. We confirm that none of the cited articles are retracted. Formatting inconsistencies have been corrected, and references have been standardized according to journal requirements.

**Responses to Editorial Comments**

**Editor Comment 1:**

The third paragraph under the introduction can be summarized using the 3 delays model by Deborah Maine.

**Response**

Thank you for this important suggestion. We have revised the third paragraph of the Introduction to explicitly frame the discussion within the Three Delays Model, clearly explaining how inadequate knowledge of obstetric danger signs contributes primarily to delays in decision-making to seek care.

**Editor Comment 2:**

Under the study population, highlight the inclusion and exclusion criteria

**Response**

We have revised the “Study Population” section to clearly present the inclusion and exclusion criteria under separate subheadings for better clarity and readability

**Editor Comment 3:**

Replace "Variable and measurement" on page 8 with "Research Instrument and measurements."

**Response**:

This has been corrected. The section now titled **“Research Instrument and measurement”** in accordance with the recommendation.

**Editor Comment 4:**

Under the discussion, interpret the findings before comparison with extant literature

**Response**:

We have revised the Discussion section to first interpret the key findings of the study before comparing them with existing literature. This improves logical flow and strengthens the analytical depth of the discussion.

**Responses to Reviewer #1**

**Comment 1:**

Clarify the calculation of sample size

**Response:**

We have clarified the sample size calculation by explicitly presenting the formula, defining all parameters (Z, p, d), and showing the calculation steps. This has been revised in the methods section.

**Comment 2:**

Explain the criteria of selection of clinic and describe the antenatal services

**Response:**

We have expanded the description of the selected health facilities in the methods section. Specifically, we clarified that the selected facilities are public Maternal and Child Health (MCH) centers, described the services provided (ANC, delivery, postnatal care, health education, laboratory services), and explained that they follow standardized protocols and are staffed by trained healthcare professionals. The rationale for selection and representativeness has also been clarified.

**Comment 3:**

What is the definition of good, low knowledge level? and based on which criteria or KPI?

**Response:**

We have clearly defined “good knowledge” and poor knowledge in the Methods section. The classification is based on the ability of respondents to spontaneously mention at least three key obstetric danger signs across pregnancy, childbirth, and postpartum periods., in line with the JHPIEGO maternal health framework and previous literature. This has now been explicitly explained.

**Responses to Reviewer #2**

**Comment 1:**

Spelling and grammatical errors need correction

**Response**:

We have carefully reviewed the entire manuscript and corrected all spelling and grammatical errors. The manuscript has been thoroughly edited to ensure clarity, consistency, and adherence to standard academic English.

**Comment 2:**

What did you consider as being “critically ill”?

**Response**:

Thank you for this important clarification. We have revised the manuscript to explicitly define “critically ill” participants in the Methods section. In this study, critically ill pregnant women were defined as those experiencing severe medical or obstetric conditions that impaired their ability to communicate or safely participate in an interview. This clarification has been added under the exclusion criteria.

**Comment 3:**

Does parity as used here refer to total pregnancies including miscarriages or to births only?

**Response**:

We appreciate this important observation. It was pure typographic error. It was corrected and clearly stated in the Result section.

**Comment 4:**

The age at first marriage range categorisation is not of uniform interval.

**Response**:

Thank you for highlighting this issue. We have revised the categorization of age at first marriage to ensure uniform interval grouping. The categories have been adjusted to consistent intervals to improve clarity and comparability of the data.

**Comment 5:**

Similarly, age at first pregnancy range intervals is not uniform.

**Response**:

We appreciate this observation. The categorization of age at first pregnancy has been revised to ensure consistent interval groupings across categories. This change has been made in the Result section (Table 2).

**Comment 6:**

Considering the limitations of the study, the word “alarmingly” should not be used.

**Response**:

Thank you for this valuable suggestion. We agree that the term alarmingly” may overstate the findings given the study design. The word has been removed.

**Comment 7:**

This reference is the same as reference number 1.

**Response**:

Thank you for pointing out this duplication. We have carefully reviewed the reference list and identified the repeated citation. The duplicate reference has been removed, and the reference list has been updated accordingly. All in-text citations have been checked and renumbered to ensure consistency and accuracy throughout the manuscript.

**Conclusion**

We believe that the manuscript has been significantly improved the Editor’s and reviewers’ comments. We sincerely appreciate the time and effort invested in reviewing our work and hope that the revised manuscript meets the journal’s expectations.

Thank you for your consideration.

**Sincerely**,

Abdeta Muktar Ahmed (On behalf of all authors)
