# Supplementary material for: Maternal knowledge of obstetric danger signs and related factors among antenatal care attendees in Hargeisa, Somaliland: A cross-sectional analysis
Source: PLoS One. 2026 Jul 15;21(7):e0353778. doi: 10.1371/journal.pone.0353778 (PMC13372116; doi:10.1371/journal.pone.0353778)
Supplement: Attachment — Submitted filename: PONE-D-25-51252 OBSTETRIC DANGER SIGNS.docx [file pone.0353778.s003.docx]

PLOS ONE

Maternal Knowledge of Obstetric Danger Signs and Related Factors among Antenatal Care Attendees in Hargeisa, Somaliland: A Cross-Sectional Analysis

--Manuscript Draft--

| Manuscript Number: | PONE-D-25-51252 |
| --- | --- |
| Article Type: | Research Article |
| Full Title: | Maternal Knowledge of Obstetric Danger Signs and Related Factors among Antenatal Care Attendees in Hargeisa, Somaliland: A Cross-Sectional Analysis |
| Short Title: | Knowledge of Obstetric Danger Signs |
| Corresponding Author: | Abdeta Muktar Ahmed  Addis Ababa Medical University College Hargeisa Campus Hargeisa, Somaliland SOMALIA |
| Keywords: | Obstetric danger signs; knowledge and awareness; Antenatal care; pregnancy complications; Hargeisa; Somaliland. |
| Abstract: | Background: Maternal mortality remains a major global health challenge,  disproportionately affecting low- and middle-income countries. Despite international efforts, nearly 95% of maternal deaths occur in these regions, with Sub-Saharan Africa bearing the highest burden. Limited knowledge of obstetric danger signs is a key factor contributing to delays in seeking care, which in turn increases the risk of preventable maternal complications. Evidence on maternal awareness in Somaliland is scarce. Objective: This study aimed to assess the level of knowledge of obstetric~~s~~ danger signs and associated factors among pregnant women attending antenatal care in Hargeisa, Somaliland.  Methods: A facility-based cross-sectional study was conducted from July 2 to August 4,  2022, among pregnant women attending antenatal care services. A total of 222 participants were selected using a systematic random sampling technique from eight maternal and child health centers. Data were collected through face-to-face interviews using a pretested and structured questionnaire and analyzed with SPSS version 25.  Bivariate and multivariate logistic regression was carried out.  Results: About 35.6% were knowledgeable specifically about pregnancy related obstetric danger signs. Only 57 women (25.7%) demonstrated adequate overall knowledge of obstetric danger~~s~~ signs across pregnancy, childbirth, and the postpartum periods, Knowledge was highest for vaginal bleeding (65% - 67%), while less visible complications such as convulsions and blurred vision were less frequently mentioned. Multivariate analysis revealed that secondary education or higher (AOR = 3.6, 95% CI: 1.8-7.3), access to and usage of media (AOR = 2.6, 95% CI: 1.2-5.6), and institutional delivery (AOR = 3.4, 95% CI: 1.6-7.2), were significant predictors of knowledge. Conclusion: Maternal knowledge of obstetric danger signs in Hargeisa was alarmingly low. Targeted health education, broader use of mass media, and promoting  institutional delivery are critical strategies to improve and reduce preventable maternal mortality. |
| Order of Authors: | Abdeta Muktar Ahmed |
|  | Mohamed Abdilahi Ahmed |
|  | Abdulkadir Mohammed Nuh |
|  | Hamse Khalif Hassan |
| Additional Information: |  |
| Question | Response |
| Financial Disclosure  Enter a financial disclosure statement that describes the sources of funding for the work included in this submission. Review | The author(s) received no specific funding for this work. |

| the [submission guidelines](http://journals.plos.org/plosone/s/submission-guidelines#loc-financial-disclosure-statement) for detailed requirements. View published research articles from [PLOS ONE](https://journals.plos.org/plosone/) for specific examples.  This statement is required for submission and will appear in the published article if the submission is accepted. Please make sure it is accurate. |  |
| --- | --- |
| Funded studies Enter a statement with the following details: - Initials of the authors who received each award - Grant numbers awarded to each author - The full name of each funder - URL of each funder website - Did the sponsors or funders play any role in the study design, data collection and analysis, decision to publish, or preparation of the manuscript? |  |
| Did you receive funding for this work? |  |
| Competing Interests  Use the instructions below to enter a competing interest statement for this submission. On behalf of all authors, disclose any [competing interests](http://journals.plos.org/plosone/s/competing-interests) that could be perceived to bias this work—acknowledging all financial support and any other relevant financial or nonfinancial competing interests.  This statement is required for submission and will appear in the published article if the submission is accepted. Please make sure it is accurate and that any funding sources listed in your Funding Information later in the submission form are also declared in your Financial Disclosure statement.  View published research articles from [PLOS ONE](http://journals.plos.org/plosone/) for specific examples. | The authors have declared that no competing interests exist. |

| NO authors have competing interests Enter: The authors have declared that no competing interests exist. Authors with competing interests Enter competing interest details beginning with this statement: I have read the journal's policy and the authors of this manuscript have the following competing interests: [insert competing interests here] |  |
| --- | --- |
| * typeset |  |
| Ethics Statement  Enter an ethics statement for this submission. This statement is required if the study involved:   - Human participants - Human specimens or tissue - Vertebrate animals or cephalopods - Vertebrate embryos or tissues - Field research   Write "N/A" if the submission does not require an ethics statement.  General guidance is provided below. Consult the [submission guidelines](http://journals.plos.org/plosone/s/submission-guidelines#loc-guidelines-for-specific-study-types) for detailed instructions. Make sure that all information entered here is included in the Methods section of the manuscript. | Ethical approval for the study was obtained from the Research Ethics Committee of Addis Ababa Medical University College, Hargeisa campus (Ref. No:  AAMUCHC/REC/87/22). The purpose of the study was explained to all participants, and written informed consent was obtained prior to data collection. Participation was voluntary, and respondents were assured that their information would be used solely for research purpose and kept confidential. Names and personal identifiers were not recorded to preserve anonymity. |

Format for specific study types

Human Subject Research (involving human

participants and/or tissue)

Give the name of the institutional review

board or ethics committee that approved the

study

•

Include the approval number and/or a

statement indicating approval of this

research

•

Indicate the form of consent obtained

(

written/oral) or the reason that consent was

not obtained (e.g. the data were analyzed

anonymously)

•

Animal Research (involving vertebrate

animals, embryos or tissues)

Provide the name of the Institutional Animal

Care and Use Committee (IACUC) or other

relevant ethics board that reviewed the

study protocol, and indicate whether they

approved this research or granted a formal

waiver of ethical approval

•

Include an approval number if one was

obtained

•

If the study involved

non-human primates

,

add

additional details

about animal welfare

and steps taken to ameliorate suffering

•

If anesthesia, euthanasia, or any kind of

animal sacrifice is part of the study, include

briefly which substances and/or methods

were applied

•

Field Research

Include the following details if this study

involves the collection of plant, animal, or

other materials from a natural setting:

Field permit number

•

Name of the institution or relevant body that

granted permission

•

Data Availability

Authors are required to make all data

underlying the findings described fully

available, without restriction, and from the

time of publication. PLOS allows rare

exceptions to address legal and ethical

concerns. See the

[PLOS Data Polic](http://journals.plos.org/plosone/s/data-availability)

[y](http://journals.plos.org/plosone/s/data-availability)

and

[FA](http://journals.plos.org/plosone/s/data-availability#loc-faqs-for-data-policy)

[Q](http://journals.plos.org/plosone/s/data-availability#loc-faqs-for-data-policy)

for detailed information.

Yes - all data are fully available without restriction

| A Data Availability Statement describing where the data can be found is required at submission. Your answers to this question constitute the Data Availability Statement and will be published in the article, if accepted. |  |
| --- | --- |
| Important: Stating ‘data available on request from the author’ is not sufficient. If your data are only available upon request, select ‘No’ for the first question and explain your exceptional situation in the text box. |  |
| Do the authors confirm that all data underlying the findings described in their manuscript are fully available without restriction? |  |
| Describe where the data may be found in full sentences. If you are copying our sample text, replace any instances of XXX with the appropriate details.   - If the data are held or will be held in a public repository, include URLs, accession numbers or DOIs. If this information will only be available after acceptance, indicate this by ticking the box below. For example: All XXX files   are available from the XXX database (accession number(s) XXX, XXX.).   - If the data are all contained within the manuscript and/or Supporting Information files, enter the following: All relevant data are within the manuscript and its Supporting Information files. - If neither of these applies but you are able to provide details of access elsewhere, with or without limitations, please do so. For example:   Data cannot be shared publicly because of [XXX]. Data are available from the XXX Institutional Data Access / Ethics Committee (contact via XXX) for researchers who meet the criteria for access to confidential data.  The data underlying the results presented in the study are available from (include the name of the third party | All relevant data are within the manuscript and its Supporting Information files. |

| and contact information or URL).  • This text is appropriate if the data are owned by a third party and authors do not have permission to share the data.  * typeset |  |
| --- | --- |
| Additional data availability information: |  |

Manuscript	[Click here to access/download;Manuscript;Knowledge](https://www2.cloud.editorialmanager.com/pone/download.aspx?id=42042493&guid=a068f6c3-ee22-45f1-8ba5-c052a06b2e43&scheme=1)

[PLOS.docx](https://www2.cloud.editorialmanager.com/pone/download.aspx?id=42042493&guid=a068f6c3-ee22-45f1-8ba5-c052a06b2e43&scheme=1)

**Maternal Knowledge of Obstetric Danger Signs and Related**

**Factors among Antenatal Care Attendees in Hargeisa,**

# Somaliland: A Cross-Sectional Analysis

Abdeta Muktar Ahmed ^1*^, Mohamed Abdilahi Ahmed ^1^, Abdulkadir Mohammed Nuh ^2^, Hamse Khalif Hassan ^1,3^

1 Addis Ababa Medical University College, Hargeisa campus, Department of Public Health, Hargeisa, Somaliland
2 Addis Ababa Medical University College, Hargeisa campus, Department of Nutrition, Hargeisa, Somaliland
3 International Health program, college of medicine, National Yang Ming Chiao Tung University (NYCU), Taipei city, Taiwan

* Corresponding author

E-mail: abdetamuk16@gmail.com

# Abstract

**Background**: Maternal mortality remains a major global health challenge, disproportionately affecting low- and middle-income countries. Despite international efforts, nearly 95% of maternal deaths occur in these regions, with Sub-Saharan Africa bearing the highest burden. Limited knowledge of obstetric danger signs is a key factor contributing to delays in seeking care, which in turn increases the risk of preventable maternal complications. Evidence on maternal awareness in Somaliland is scarce.

**Objective**: This study aimed to assess the level of knowledge of obstetric~~s~~ danger signs and associated factors among pregnant women attending antenatal care in Hargeisa, Somaliland.

**Methods**: A facility-based cross-sectional study was conducted from July 2 to August 4, 2022, among pregnant women attending antenatal care services. A total of 222 participants were selected using a systematic random sampling technique from eight maternal and child health centers. Data were collected through face-to-face interviews using a pretested and structured questionnaire and analyzed with SPSS version 25. Bivariate and multivariate logistic regression was carried out.

**Results**: About 35.6% were knowledgeable specifically about pregnancy related obstetric danger signs. Only 57 women (25.7%) demonstrated adequate overall knowledge of obstetric danger~~s~~ signs across pregnancy, childbirth, and the postpartum periods, Knowledge was highest for vaginal bleeding (65% - 67%), while less visible complications such as convulsions and blurred vision were less frequently mentioned. Multivariate analysis revealed that secondary education or higher (AOR = 3.6, 95% CI: 1.8-7.3), access to and usage of media (AOR = 2.6, 95% CI: 1.2-

5.6), and institutional delivery (AOR = 3.4, 95% CI: 1.6-7.2), were significant predictors of knowledge.

**Conclusion**: Maternal knowledge of obstetric danger signs in Hargeisa was alarmingly low. Targeted health education, broader use of mass media, and promoting institutional delivery are critical strategies to improve and reduce preventable maternal mortality.

**Key words**: Obstetric danger signs, knowledge and awareness, antenatal care, pregnancy complications, Hargeisa, Somaliland.

# Background

The maternal mortality remains a pressing global issue. In 2023, more than 700 women lost their lives to pregnancy-related and childbirth-related avoidable causes every day, or roughly one woman every two minutes [1]. Despite international efforts to address it, maternal mortality is still a serious global health concern. The startling truth is that the majority of maternal fatalities are avoidable and result from severe health disparities that occur both within and between nations and worldwide. These disparities are significant. Nearly 95% of all maternal deaths take place in low- and middle-income nations, with Sub-Saharan Africa bearing the highest risk, accounting for 70% of all maternal deaths. The 2030 United Nations Sustainable Development Goal (SDG), which aims to lower the rate of maternal fatalities to fewer than 70 per 100,000 live births, is still significantly above the current global rate of maternal deaths (223 per 100,000 live births) [2-3].

Even though, an estimated 260,000 maternal deaths, or about 700 deaths per day, occurred globally in 2023, maternal mortality has decreased by 40% since 2000, which is indicative of ongoing international efforts to provide access to basic healthcare services. No region was designated as having a very high maternal mortality ratio (MMR), and no country was predicted to have an exceptionally high MMR for the first time [4].

One of the main causes of maternal mortality in the underdeveloped world is women’s, families’, and birth attendants’ lack of awareness about the obstetric warning signs. These deaths can be decreased by educating women about the warning signs of obstetric~~s~~ dangers, encouraging them to seek medical attention when necessary, and providing them on counselling on these signs of obstetrics danger, as well as the right management of these complications [5]. Women should be aware of the warning signs of obstetric dangers, during pregnancy, birth, and the postpartum period, as every pregnancy faces risk. In the end, the information will enable them and their families to decide quickly to seek care from trained birth attendants [6].

Obstetric danger signs are warning indications that could point to potentially life-threatening conditions during pregnancy, labor, or the postpartum phases. About 75% of all maternal deaths during these three phases are caused by obstetric danger signs, which include vaginal bleeding, blurred vision, lack of fetal movement, a gush of fluid from the vagina, high fever, retained placenta, foul-smelling discharge, convulsions, and severe headaches. Because mothers and family members may quickly recognize these signs without need for a complex professional diagnosis, raising maternal knowledge is crucial approach in minimizing care-seeking delays [7]. Pregnant women who notice warning symptoms and seek prompt obstetric care could reduce maternal mortality from complications connected to childbirth in low-income countries. One of the most frequent reasons why people fail to detect complications when they arise and put off seeking care is a lack of warning signs [8]. Poor maternal and newborn outcomes result from pregnant women’s lack of awareness of obstetrics risk signs, which is a major cause in delays in seeking care. Designing focused treatments that raise awareness, encourage prompt care-seeking behavior, and improve birth preparedness requires an understanding of the factors influencing pregnant women’s knowledge of obstetric~~s~~ danger signs [9].

According to the 2020 Somaliland Health and Demographic Survey (SLHDS), the country experiences an estimated 396 maternal deaths for every 100,00 live births. Furthermore, only

47% of pregnant women reported attending at least one antenatal care (ANC) visit, and merely 33% delivered with the assistance of skilled birth attendant [10]. Although Somaliland’s maternal mortality ratio has shown a declining trend and is not among the highest in the region, it still remains far from achieving the SDG target of reducing maternal mortality to 70 per 100,000 live births by 2030.

To date, no studies have been conducted in Hargeisa, Somaliland, to assess the level of knowledge regarding obstetric danger signs and their associated factors. Understanding this knowledge gap is crucial, as improving awareness of obstetric danger signs can inform strategies to enhance maternal and health outcomes and reduce maternal mortality. Therefore, the present study aims to evaluate the level of knowledge of obstetric danger signs and identify the associated factors among pregnant women attending antenatal care (ANC) services in selected health institutions in Hargeisa, Somaliland.

# Materials and Methods

## Study design and area

A facility-based cross-sectional study was conducted in Hargeisa, the capital of Somaliland, from July 2 to August 4, 2022. Somaliland, a self-declared autonomous state in the horn of Africa that unilaterally separated from Somalia in 1991, had an estimated population of 6.2 million [11].

Hargeisa, its capital, is home to approximately 1.2 million people [12].

## Study population

The source population consisted of pregnant women attending ANC services at health institutions in Hargeisa during the study period. All pregnant women who were physically able and willing to participate were included, whereas those who were critically ill at the time of data collection were excluded.

The sample size was calculated using a single population proportion formula, taking into account of a 15.5% [13] prevalence of knowledge of obstetrics danger signs from a previous study, a 5% margin of error, 95% confidence level, and a 10% allowance for non-response. The final sample size was 222. Out of the 17 Maternal and Child Health (MCH) centers in Hargeisa, eight were selected through simple random sampling. Proportional allocation was applied to distribute the study units across the selected facilities, based on the number of ANC attendees recorded in the month preceding the survey. The sampling interval (K) was calculated as about three, meaning every third eligible woman attending ANC was invited to participate until the allocated quota for each facility was reached.

## Variables and measurement

Data were collected using pretested, structured, interviewer-administered questionnaire adapted from the Maternal and Newborn Health Program of JHPIEGO, and affiliate of Johns Hopkins

University and other related articles [14-15]. The tool comprised four sections: (1)

Sociodemographic characteristics, (2) Reproductive and obstetric~~s~~ history, (3) Service utilization, and (4) Knowledge of obstetric~~s~~ danger signs. The questionnaire was initially prepared in English, translated into Somali, and subsequently back-translated into English to ensure consistency and accuracy. Necessary revisions were made based on feedback from the pretest.

Women’s knowledge of obstetric danger signs was assessed by asking participants to spontaneously mention danger signs that may occur during pregnancy, childbirth, and the postpartum period, which were categorized into three groups. Knowledge of pregnancy danger signs (the study’s dependent variable) was defined as correctly identifying at least three key signs, including vaginal bleeding, swollen hands/face, and blurred vision. Knowledge of childbirth/labor danger signs was defined as mentioning at least three major signs, namely vaginal bleeding, prolonged labor, convulsions, or retained placenta. For the postpartum period, women were regarded knowledgeable if they identified at least three major signs, including vaginal bleeding after delivery, loss of consciousness, and postpartum fever. Overall, a woman was considered **knowledgeable on obstetric~~s~~ danger signs** if she was able to mention at least three key signs across each of the three phases, without prompting [15]. For analysis, the dependent variables were dichotomized into “**knowledgeable**” and “**not knowledgeable**.”

## Data collection procedures

Data were collected through face-to-face interview conducted by six trained BSc Nursing students. Two experienced supervisors oversaw the process. Prior to data collection, the questionnaire was pretested on 22 pregnant women at an MCH center outside the study area. Based on pretest findings, adjustments were made to improve clarity and accuracy. Training was provided to data collectors and supervisors on the objectives of the study, the data collection tool, ethical considerations, and procedures to ensure confidentiality.

## Data processing and analysis

Completed questionnaires were checked daily for completeness and consistency. Data were cleaned, coded, and entered into SPSS version 25 for analysis. Descriptive statistics were used to summarize the data. Bivariate logistic regression was first performed, and variables with a p-value < 0.2 were considered candidates for multivariate analysis. Subsequently, multivariate logistic regression was carried out to examine associations between independent variables and the outcome variable while controlling for potential confounding factors. Adjusted odds ratios (AOR) with 95% confidence interval (CI) were computed to determine the strength of associations. Statistical significance was declared at p<0.05.

## Ethical considerations

Ethical approval for the study was obtained from the Research Ethics Committee of Addis Ababa Medical University College, Hargeisa campus (Ref. No: AAMUCHC/REC/87/22). The purpose of the study was explained to all participants, and written informed consent was obtained prior to data collection. Participation was voluntary, and respondents were assured that their information would be used solely for research purpose and kept confidential. Names and personal identifiers were not recorded to preserve anonymity.

# Result

## Socio-demographic characteristics

A total of 222 pregnant women participated in the study, yielding a response rate of 100%. The mean age of the respondents was 28.3 years (±6.5 SD), with a median age of 28.5 years, ranging from 17 to 43 years. More than half of the respondents (55.4%) were between 25 and 36 years of age. Most participants (201, 90.5%) were currently married. Regarding educational attainment, 75 (33.8%) had no formal education, 53 (23.9%) had completed primary education, 38 (17.1%) had secondary education, and 56 (25.2%) attained higher education. By contrast, more than half of their spouses (121, 54.5%) had higher education, while only 59 (26.6%) had primary or no formal education. In terms of occupation, the majority of respondents (153, 68.9%) were merchants, 49 (22.1%) were housewives/unemployed, and 20 (9%) were formally employed. Among spouses, 149 (67.1%) were employed, 62 (27.9%) were merchants, and 11 (5%) were unemployed. The mean monthly household income was 561 USD. A large proportion (152, 68.5%) reported monthly incomes of ≤500 USD. Additionally, 142 respondents (64%) had access to mainstream or social media (Table 1).

### Table 1. Socio-demographic characteristics of the respondents (n=222)

| **Variables** | **Category** | **Frequency (n)** | **Percentage (%)** |
| --- | --- | --- | --- |
| Age (years) | ≤24 | 70 | 31.5 |
|  | 25-36 | 123 | 55.4 |
|  | ≥37 | 29 | 13.1 |
| Marital status | Currently married | 201 | 90.5 |
|  | Widowed/Divorced | 21 | 9.5 |
| Educational status  (women) | No formal education | 75 | 33.8 |
|  | Primary | 53 | 23.9 |
|  | Secondary | 38 | 17.1 |
|  | Higher education | 56 | 25.2 |
| Educational status  (spouse) | No formal education | 36 | 16.2 |
|  | Primary | 23 | 10.4 |
|  | Secondary | 42 | 18.9 |
|  | Higher education | 121 | 54.5 |
| Occupation (women) | Merchant | 153 | 68.9 |
|  | Housewife/Unemployed | 49 | 22.1 |
|  | Employed | 20 | 9 |
| Occupation (Spouse) | Employed | 149 | 67.1 |
|  | Merchant | 62 | 27.9 |
|  | Unemployed | 11 | 5 |
| Monthly family income (USD) | ≤500 | 152 | 68.5 |
|  | 501-1000 | 30 | 13.5 |
|  | >1000 | 40 | 18 |
| Access to media | Yes | 142 | 64 |
|  | No | 80 | 36 |

## Obstetric~~s~~ characteristics

The age at first marriage ranged from 14 to 28 years, with a mean of 18.9 years. Half of the respondents (111, 50%) married between the age of 14-18 years. The age at first pregnancy ranged from 16 to 30 years, with a mean of 20 years; more than half (119, 53.6%) conceived between 19-24 years. At the time of data collection, nearly half of the respondents (102, 45.9%) were in their third trimester.

Parity ranged from 1 to 11 pregnancies, with a mean of 4.5. Among them, 95 (42.8%) had 4-6 pregnancies, and 136 (61.3%) reported up to three births. Regarding pregnancy intention, 144 (64.9%) reported their current pregnancy was planned, while 78 (35.1%) stated otherwise. A majority of the participants (178, 80.2%) reported not experiencing any obstetric~~s~~ danger signs during their current pregnancy (Table 2).

### Table 2. Obstetric variables of the study participants (n=222)

| **Variables** | **Category** | **Frequency (n)** | **Percent (%)** |
| --- | --- | --- | --- |
| Age at first marriage  (years) | 14-18 | 111 | 50 |
|  | 19-24 | 98 | 44.1 |
|  | ≥25 | 13 | 5.9 |
| Age at first pregnancy (years) | 16-18 | 80 | 36 |
|  | 19-24 | 119 | 53.6 |
|  | ≥25 | 23 | 10.4 |
| Trimester (Current pregnancy) | First | 54 | 24.5 |
|  | Second | 66 | 29.7 |
|  | Third | 102 | 45.9 |
| Number of pregnancies | ≤3 | 83 | 37.4 |
|  | 4-6 | 95 | 42.8 |
|  | >6 | 44 | 19.8 |
| Number of births | ≤3 | 136 | 61.3 |
|  | 4-6 | 62 | 27.9 |
|  | >6 | 24 | 10.8 |
| Current pregnancy intended | Yes | 144 | 64.9 |
|  | No | 78 | 35.1 |
| History of danger signs | Yes | 44 | 19.8 |
|  | No | 178 | 80.2 |

## Maternal Health Service Utilization

With respect to ANC visits during the current pregnancy, 63 women (28.4%) had attended only once, while 66 (29.7%), 55 (24.8%), and 38 (17.2%) had attended two, three, and four or more visits, respectively. More than two-thirds, (158, 71.2%), had received health education about obstetric danger signs during ANC visits, whereas 64 (28.8%) had not.

Among 209 respondents with previous childbirth experience, 128 (61.2%) delivered their last baby in a health institution, while 81 (38.8%) delivered at home. The majority, (187, 89.5%) reported spontaneous vaginal delivery, followed by instrumental delivery, (15, 7.2%), and cesarean section (7, 3.3%).

The reported travel time to the nearest health facility ranged from 1 to 120 minutes, with a mean of 33 minutes. More than half (130, 58.6%) lived within a 30-minutes walking distance.

Regarding decision-making autonomy, 116 (52.3%) reported making decisions independently, 82 (36.9%) jointly with their partner, and 24 (10.8%) reported that decisions were solely by their partner. Only 85 (38.3%) discussed maternal health service utilization with their spouses, while the majority, (137, 61.7%) did not (Table 3).

### Table 3. Maternal health service utilization (n=222)

| **Variables** | **Category** | **Frequency (n)** | **Percentage (%)** |
| --- | --- | --- | --- |
| ANC visits (current pregnancy) | 1-3 | 184 | 82.9 |
|  | ≥4 | 38 | 17.2 |
| Received health education on danger signs | Yes | 158 | 71.2 |
|  | No | 64 | 28.8 |
| Place of last delivery  (n=209) | Home | 81 | 38.8 |
|  | Health institution | 128 | 61.2 |
| Mode of delivery (n=209) | Spontaneous vaginal delivery (SVD) | 187 | 89.5 |
|  | Instrumental Delivery | 15 | 7.2 |
|  | Cesarean section | 7 | 3.3 |
| Time to nearest health facility (walk) | ≤30 min | 130 | 58.6 |
|  | >30 min | 92 | 41.4 |
| Decision making power | Self | 116 | 52.3 |
|  | Joint (both) | 82 | 36.9 |
|  | Partner only | 24 | 10.8 |
| Discussion with spouse on maternal services | Yes | 85 | 38.3 |
|  | No | 135 | 61.7 |

## Knowledge of obstetric Danger Signs

Respondents were asked to list key obstetric~~s~~ danger signs spontaneously. During pregnancy, the most commonly mentioned signs were vaginal bleeding (144, 64.9%), blurred vision (126,

56.8%), swollen face and limbs (119, 53.6%), severe headache (108, 48.6%), hypertension (75,

33.8%), and convulsions (73, 32.9%).

During childbirth, prolonged labor, 150 (67.6%), and vaginal bleeding, 149 (67.1%), were the most frequently mentioned danger signs, followed by retained placenta 115 (51.8%), hypertension, 96 (43.2%), and convulsions, 92 (41.4%).

In the postnatal period, vaginal bleeding, 145 (65.3%), was the most frequently mentioned sign, followed by high fever, 131 (59%), foul-smelling vaginal discharge, 98 (44.1%), severe weakness, 93 (41.9%), and difficulty breathing, 83 (37.4%) (Table 4).

### Table 4. Knowledge of obstetric danger signs mentioned by the respondents (n=222)

### (a) During pregnancy

| **Danger sign** | **Frequency (n)** | **Percent (%)** |
| --- | --- | --- |
| Vaginal bleeding | 144 | 64.9 |
| Blurred vision | 126 | 56.8 |
| Swollen face/limbs | 119 | 53.6 |
| Severe headache | 108 | 48.6 |
| Hypertension | 75 | 33.8 |
| Convulsions | 73 | 32.9 |
| Persistent nausea /vomiting | 71 | 32 |
| Malpresentation/position | 71 | 32 |
| Reduced/absent fetal movement | 68 | 30.6 |
| Severe abdominal cramp | 66 | 29.7 |
| Leakage of amniotic fluid | 46 | 20.7 |

### (b) During childbirth

| **Danger sign** | **Frequency (n)** | **Percent (%)** |
| --- | --- | --- |
| Prolonged labor | 150 | 67.6 |
| Vaginal bleeding | 149 | 67.1 |
| Retained placenta | 115 | 51.8 |
| Hypertension | 96 | 43.2 |
| Convulsions | 92 | 41.4 |
| Cessation of labor pain | 81 | 36.5 |
| High fever | 80 | 36 |
| Swollen face/limbs | 74 | 33.3 |
| Severe headache | 71 | 32 |
| Malpresentation/position | 59 | 26.6 |
| Blurred vision | 52 | 23.4 |
| Severe abdominal cramps | 46 | 20.7 |

### (c) During postnatal period

| **Danger sign** | **Frequency (n)** | **Percent (%)** |
| --- | --- | --- |
| Vaginal bleeding | 145 | 65.3 |
| High fever | 131 | 59 |
| Foul-smelling vaginal discharge | 98 | 44.1 |
| Severe weakness | 93 | 41.9 |
| Difficulty breathing | 83 | 37.4 |
| Swollen face/limbs | 73 | 32.9 |
| Severe headache | 61 | 27.6 |
| Unconsciousness | 58 | 26.1 |
| Uterine prolapse | 50 | 22.5 |
| Blurred vision | 48 | 21.6 |
| Convulsions | 45 | 20.3 |

Overall, only 79 respondents (35.6%) demonstrated adequate knowledge of obstetric~~s~~ danger signs during pregnancy, which was the study’s dependent variable. Knowledge of danger signs was lower during childbirth, with 66 respondents (29.7%) identified as knowledgeable, and during the postnatal period, with 72 respondents (32.4%). When considering knowledge across all the three periods, only 57 respondents (25.7%) demonstrated adequate overall knowledge of obstetric danger signs. Notably, 35 participants (15.8%) were unable to mention even one of the three key danger signs (Figure 1).

**Figure 1. Knowledge of obstetric danger signs during pregnancy, childbirth, and postpartum periods (n=222)**

## Factors Associated with Knowledge of Obstetric Danger Signs

Bivariate and multivariate logistic regression analyses were conducted to identify factors associated with knowledge of key obstetric~~s~~ danger signs during pregnancy. In the bivariate analysis, women’s educational status, access to media, place of delivery, decisions-making autonomy, and receipt of health education during ANC were significantly associated. In the multivariate analysis, three factors remained significant predictors: women’s education level, access to media, and place of delivery.

Women with secondary education or higher were 3.6 times more likely to be knowledgeable compared to those with primary education or less (AOR = 3.6, 95% CI: 1.8-7.3, p < 0.001). Access to mainstream or social media was also significant; women with such access were 2.6 times more likely to be knowledgeable (AOR = 2.6, 95% CI: 1.2-5.6, p = 0.016). Similarly, women who delivered their last child in a health facility were 3.4 times more likely to be knowledgeable compared to those who delivered at home (AOR = 3.4, 95% CI: 1.6-7.2, p =

0.001) (Table 5).

### Table 5. Factors associated with knowledge of obstetric danger signs (n=222)

| **Variable** | **Category** | **Knowledgeable n (%)** | **Not knowledgeable n (%)** | **COR (95%**  **CI)** | **AOR (95%**  **CI)** |
| --- | --- | --- | --- | --- | --- |
| Education | Primary or less | 25 (31.6%) | 103 (72%) | 1 | 1 |
|  | Secondary or more | 54 (68.4%) | 40 (28%) | 5.5 (3.0-10.1) p<0.001 | 3.6 (1.8-7.3) p<0.001 |
| Access to media | Yes | 66 (83.5%) | 76 (53.1) | 4.5 (2.3-8.82) p<0.001 | 2.6 (1.2-5.6) p=0.016 |
|  | No | 13 (16.5%) | 67 (46.9%) | 1 | 1 |
| Place of last delivery | Home | 13 (17.1%) | 68 (51.1%) | 1 | 1 |
|  | Hospital | 63 (82.9%) | 65 (48.9%) | 5.0 (2.5- 10) p<0.001 | 3.4 (1.6-7.2) p<0.001 |

# Discussion

This study assessed maternal knowledge of obstetric~~s~~ danger signs among women attending antenatal care in Hargeisa, Somaliland, and identified key socio-demographic and service relate predictors.

The findings revealed that knowledge of obstetric danger signs during pregnancy was low, with only slightly more than one-third of women (35.6%) classified as knowledgeable. This result aligns with studies conducted in Debre Tabor, Ethiopia (39%) [6]; Jigjiga, Ethiopia (37.9%) [16]; and Zambia (35%) [9], where awareness of obstetric danger signs remained insufficient despite the implementation of maternal health programs. Similarly, lower levels of knowledge were reported in Dire Dawa, Ethiopia (24.1%) [5] and Southern Nigeria (24.9%) [17]. The relatively low knowledge observed in this study may be attributed to limited health education coverage, cultural norms influencing communication about reproductive health, and gaps in community based health promotion.

The findings revealed substantial gaps in awareness, particularly concerning pregnancy-related complications. While a proportion of respondents were able to identify one or more key danger signs, alarmingly, considerable number of women were unable to mention **any**. This gap in knowledge raises serious public health concerns, as unawareness of warning symptoms may delay timely health-seeking behavior and increase the risk of adverse maternal outcomes. Additionally, these women would be at higher risk of failing to seek emergency care promptly if complications arise. Similar patterns have been documented in resource-limited settings, such as in Bhutan [18] and in a nationwide study conducted in the Democratic Republic of Congo (DRC) [19], where women often rely on traditional beliefs, family influence, or wait until compilations worsen before deciding to seek care. The lack of knowledge among these women reflects missed opportunities for health education during routine antenatal visits, which are intended to serve not only as check-ups but also as platforms for prevention counselling.

Despite moderate ANC utilization, the number of women achieving the recommended four visits was optimal. Furthermore, in our study, spousal communication regarding maternal health was notably low (38.3%), a factor that may hinder timely decision-making during emergencies, in contrast to male involvement promoting positive maternal health service utilization. A systematic review and meta-analysis from low- and middle-income countries likewise positively identified male involvement as a positive factor to maternal health-seeking behavior including knowledge of obstetric danger signs. [20].

Vaginal bleeding emerged as the most commonly recognized danger sign across pregnancy, childbirth, and the postpartum period. This finding aligns with studies in Shashamene, Ethiopia [8], Cameroon [21], eastern DRC [22], and Rwanda [23], as well as with a systematic review from developing countries [24], where bleeding was consistently the most frequently recognized danger sign, likely due to its obvious and visible nature. In contrast, danger signs such as convulsion and blurred vision were less commonly mentioned, highlighting knowledge gaps in recognizing less visible but equally life-threatening complications like pre-eclampsia and eclampsia.

Educational status appeared to play an important role in knowledge levels, as women with secondary or higher education were more likely to correctly identify danger signs than their less educated counterparts with little or no schooling. This finding aligns with evidence from other studies conducted in Bahir Dar, Ethiopia [15], Cameroon [21], India [25], Indonesia [26], southern Nigeria [17], and Tanzania [27], where literacy was positively associated with maternal knowledge and health seeking behavior. This underscores the critical role of female education in enhancing maternal health outcomes.

Access to and use of media also played a crucial role, suggesting that health promotion through radio, television, and social media platforms can substantially improve maternal awareness. This finding is consistent with studies in Jigjiga, Ethiopia [16], and Indonesia [26], where mass media health education was proved effective in enhancing health literacy among women of reproductive age. Such media exposures and campaigns not only increased awareness of obstetric danger signs but also encouraged greater utilization of community-based interventions to bridge knowledge gaps and promote positive health-seeking behavior. These results highlight the critical role of accessible information channels in shaping maternal health outcomes, particularly in settings where formal educations levels may be relatively low.

Institutional delivery was another strong predictor of knowledge, as women who delivered in health facilities were more likely to recall danger signs, possibly due to counselling received during delivery and postnatal care. This finding supports WHO recommendations emphasizing skilled birth attendance as a key avenue for maternal health education. Furthermore, evidence from a systematic review in developing counties [24], and a study conducted in Zambia [9] support this association, highlighting the role of facility-based care in improving women’s awareness.

Overall, the results suggest that while some progress has been made, substantial gaps remain in maternal knowledge, particularly concerning less obvious danger signs and across the pregnancy, childbirth and postnatal periods.

## Strengths and limitations

The study provides context-specific evidence on maternal knowledge of obstetric danger signs, which is essential for designing targeted interventions. In addition, the use of a structured questionnaire allowed for systematic assessment of knowledge across multiple domains. However, the following limitations should be considered. The cross-sectional design precludes establishing cause-and-effect relationships. Self-reported knowledge may be subject to recall or social desirability bias. Furthermore, the study was limited to women attending health facilities, which may not represent those who do not access ANC services and who might have even lower awareness. Despite its limitations, the study provides valuable evidence for public health by identifying gaps in maternal knowledge of obstetric danger signs.

## Conclusion

This study demonstrated that knowledge of obstetric danger signs among pregnant women in Hargeisa is alarmingly low, with only about one-third of the respondents knowledgeable about danger signs during pregnancy and barely one-quarter demonstrating overall adequate knowledge. It highlights critical gaps in maternal knowledge, with a particular concerning proportion of women unable to identify any key warning signs. Vaginal bleeding was the most commonly recognized danger sign, but awareness of other critical signs was limited. Maternal education, access to media, and institutional delivery were identified as key determinants of knowledge. Therefore, designing enhanced and targeted health education programs, and facility-based interventions can help bridge the critical gaps.

## Acknowledgements

We would like to express out heartfelt gratitude to Addis Ababa Medical University College

(AAMUC), Hargeisa Campus, for their invaluable support and cooperation throughout this study. Our deepest appreciation goes to our supervisors for their continuous guidance and encouragement. We are also sincerely thankful to the data collectors and research team members for their dedication and commitment. Finally, we extend our profound gratitude to the mothers who participated in this study; without their willingness and contribution, this research would not have been possible.

# Reference

1. World Health Organization. Trends in maternal mortality 2000 to 2023: estimates by WHO, UNICEF, UNFPA, World Bank Group and UNDESA/Population Division. [https://www.who.int/publications/i/item/9789240108462.](https://www.who.int/publications/i/item/9789240108462) Accessed on April 2025.

**2.** Wilson center. Global Health and Gender Policy Brief: Drivers of Global Maternal Mortality. [https://www.wilsoncenter.org/publication/global-health-and-gender-policybrief-drivers-global](https://www.wilsoncenter.org/publication/global-health-and-gender-policy-brief-drivers-global-maternal-mortality#:~:text=These%20inequalities%20are%20profound.,classified%20as%20late%20maternal%20deaths) [maternalmortality#:~:text=These%20inequalities%20are%20profound.,classified%20as%20late%20maternal%20deaths.](https://www.wilsoncenter.org/publication/global-health-and-gender-policy-brief-drivers-global-maternal-mortality#:~:text=These%20inequalities%20are%20profound.,classified%20as%20late%20maternal%20deaths) Accessed on December 2024.

3. UNDP. Sustainable Development Goal (SDG). Goal 3: Good health and well-being. [https://www.undp.org/sustainable-development-goals/good-health.](https://www.undp.org/sustainable-development-goals/good-health) Accessed on March 2025.
4. UNFPA. Trends in Maternal Mortality 2000-2023: Estimates by WHO, UNICEF,

UNFPA, World Bank Group and UNDESA/Population Division. [https://www.unfpa.org/publications/trends-maternal-mortality-2000-](https://www.unfpa.org/publications/trends-maternal-mortality-2000-2023#:~:text=While%20maternal%20mortality%20spiked%20during,to%20the%20care%20they%20need)

[2023#:~:text=While%20maternal%20mortality%20spiked%20during,to%20the%20care](https://www.unfpa.org/publications/trends-maternal-mortality-2000-2023#:~:text=While%20maternal%20mortality%20spiked%20during,to%20the%20care%20they%20need)

[%20they%20need.](https://www.unfpa.org/publications/trends-maternal-mortality-2000-2023#:~:text=While%20maternal%20mortality%20spiked%20during,to%20the%20care%20they%20need) Accessed on March 2025.

5. Abdurashid N, Ishaq N, Ayele K, Ashenafi N. Level of Awareness on Danger Signs During Pregnancy and Associated Factors, among Pregnant Mothers, Dire Dawa Administrative Public Health Facilities, Eastern Ethiopia. Clinics Mother Child Health 15: 290. doi:10.4172/2090-7214.1000290.
6. Mestawut Mihret, Hailegebriel Wondimu. Assessment of Knowledge about Obstetric

Danger Signs and Associated Factors among Pregnant Women in Debre Tabor Town,

Northwest Ethiopia. Wiley online Library, Journal of Pregnancy. 2023.

[https://doi.org/10.1155/2023/1475500.](https://doi.org/10.1155/2023/1475500)

7. Bolanko A, Namo H, Minsamo K, Addisu N, Gebre M. Knowledge of obstetric danger signs and associated factors among pregnant women in Wolaita Sodo town, South Ethiopia: A community-based cross-sectional study. *SAGE Open Medicine*. 2021;9. doi:[10.1177/20503121211001161.](https://doi.org/10.1177/20503121211001161)
8. Wassihun, B., Negese, B., Bedada, H. *et al.* Knowledge of obstetric danger signs and associated factors: a study among mothers in Shashamane town, Oromia region,

Ethiopia. *Reprod Health* **17**, 4 (2020). [https://doi.org/10.1186/s12978-020-0853-z.](https://doi.org/10.1186/s12978-020-0853-z)

9. Nyirenda M, Hazemba AN, Makasa M. Determinants of knowledge of obstetric danger signs among women attending antenatal care in Eastern province, Zambia: a cross sectional study. BMC Pregnancy Childbirth. 2025 Jul 3;25(1):719. doi: 10.1186/s12884-

025-07782-y. PMID: 40610952; PMCID: PMC12224364.

10. The Somaliland Health and Demographic Survey 2020 HDS; October 2022; [https://somalia.unfpa.org/sites/default/files/pub-pdf/slhds2020_report_2020.pdf.](https://somalia.unfpa.org/sites/default/files/pub-pdf/slhds2020_report_2020.pdf)Accessed on March 2025.
11. Wikipedia, Somaliland. [https://en.wikipedia.org/wiki/Somaliland.](https://en.wikipedia.org/wiki/Somaliland) Accessed on March

2025.

12. Wikipedia, Hargeisa. [https://en.wikipedia.org/wiki/Hargeisa.](https://en.wikipedia.org/wiki/Hargeisa) Accessed on March 2025.
13. Maseresha et al. Knowledge of obstetric danger signs and associated factors among pregnant women in Erer district, Somali region, Ethiopia, BMC Women's Health. 2016;

16:30.

14. JHPIEGO. *Monitoring Birth preparedness and complication readiness: Tools Indicators for Maternal and Newborn Health*. Baltimore, Maryland: JHPIEGO Maternal and Neonatal Health Program, 2004.
15. Jewaro, M., Yenus, H., Ayanaw, Y. *et al.* Knowledge of obstetric danger signs and associated factors among mothers in Bahir Dar district, northwest Ethiopia: an institutionbased cross-sectional study. *Public Health Rev* **41**, 14 (2020).

[https://doi.org/10.1186/s40985-020-00132-7.](https://doi.org/10.1186/s40985-020-00132-7)

16. Haile, S., Mekonen, L., Shiferaw, Z., Degefu, A., Getachew, N., Alemayehu, Y., & Issak, D. (2024). Knowledge about Obstetric Danger Signs and Associated Factors among

Pregnant Mothers in Jigjiga City, Somali Regional State, Eastern, Ethiopia. *East African*

*Journal of Pastoralism*, *1*(2). Retrieved from [https://eajp.et/index.php/jju/article/view/37.](https://eajp.et/index.php/jju/article/view/37)

17. Okoror, C. E. M., & Omuemu, V. O. (2021). Knowledge of obstetric danger signs among antenatal clinic attendees in South–South Nigeria. *Health Care for Women*

*International*, *44*(10–11), 1363–1378. [https://doi.org/10.1080/07399332.2021.1941025.](https://doi.org/10.1080/07399332.2021.1941025)

18. Tamang, S.T., Dorji, T., Yoezer, S. *et al.* Knowledge and understanding of obstetric danger signs among pregnant women attending the antenatal clinic at the National

Referral Hospital in Thimphu, Bhutan: a cross-sectional study. *BMC Pregnancy*

*Childbirth* **21**, 104 (2021). [https://doi.org/10.1186/s12884-021-03580-4.](https://doi.org/10.1186/s12884-021-03580-4)

19. Nkamba, D.M., Wembodinga, G., Bernard, P. *et al.* Awareness of obstetric danger signs among pregnant women in the Democratic Republic of Congo: evidence from a nationwide cross-sectional study. *BMC Women's Health* **21**, 82 (2021). [https://doi.org/10.1186/s12905-021-01234-3.](https://doi.org/10.1186/s12905-021-01234-3)
20. Dedih Suandi, Pauline Williams, Sohinee Bhattacharya, Does involving male partners in antenatal care improve healthcare utilisation? Systematic review and meta-analysis of the published literature from low- and middle-income countries, *International Health*,

Volume 12, Issue 5, September 2020, Pages 484–

498, [https://doi.org/10.1093/inthealth/ihz073.](https://doi.org/10.1093/inthealth/ihz073)

21. Agbor Nathan Emeh et al. Antenatal care and determinants of obstetric danger signs awareness of immediate postpartum women at Buea Regional Hospital, Cameroon. Pan

African Medical Journal. 2021;38:247. [doi: [10.11604/pamj.2021.38.247.20977]](https://doi.org/10.11604/pamj.2021.38.247.20977).

22. Imani Ramazani B-E, Mabakutuvangilanga Ntala S-D, Katuashi Ishoso D, RothanTondeur M. Knowledge of Obstetric Danger Signs among Pregnant Women in the

Eastern Democratic Republic of the Congo. *International Journal of Environmental*

*Research and Public Health*. 2023; 20(8):5593. [https://doi.org/10.3390/ijerph20085593.](https://doi.org/10.3390/ijerph20085593)

23. Uwiringiyimana E, Manirambona E, Byiringiro S, Nsanzimana A, Uhawenayo N, Ufitinema P, et al. (2022) Pregnant women’s knowledge of obstetrical danger signs: A cross-sectional survey in Kigali, Rwanda. PLOS Glob Public Health 2(11): e0001084. [https://doi.org/10.1371/journal.pgph.0001084.](https://doi.org/10.1371/journal.pgph.0001084)
24. Yunitasari, E., Matos, F., Zulkarnain, H. *et al.* Pregnant woman awareness of obstetric danger signs in developing country: systematic review. *BMC Pregnancy Childbirth* **23**, 357 (2023). [https://doi.org/10.1186/s12884-023-05674-7.](https://doi.org/10.1186/s12884-023-05674-7)
25. Bhavana, B M; Yogesh, M; Padhiyar, Navneet; Damor, Naresh. Obstetric danger signs in context: A mixed methods study exploring knowledge and sociocultural factors among pregnant women. Journal of Education and Health Promotion 13(1):491, December 2024.

| DOI: 10.4103/jehp.jehp_315_24.

26. Wulandari RD, Laksono AD (2020) Determinants of knowledge of pregnancy danger signs in Indonesia. PLoS ONE 15(5): e0232550.

[https://doi.org/10.1371/journal.pone.0232550.](https://doi.org/10.1371/journal.pone.0232550)

27. Sungwa, E. E., Mwakanyamale, A., & Magesa, R. (2023). Assessment of knowledge on the danger signs of pregnancy among pregnant women at Sinza Palestina Hospital in Ubungo Municipality, Tanzania. *Tanzania Journal of Health Research*, *24*(4), 471–482.

[https://doi.org/10.4314/thrb.v24i4.](https://doi.org/10.4314/thrb.v24i4)

# Supporting information

## S1_File_SPSS_Knowledge_ODS_PLOS (SAV)

Figure	[Click here to access/download;Figure;Fig 1.tif](https://www2.cloud.editorialmanager.com/pone/download.aspx?id=42035681&guid=5de0805e-db4d-4735-b9d6-be368e4dcd04&scheme=1)

SPSS dataset

[Click here to access/download](https://www2.cloud.editorialmanager.com/pone/download.aspx?id=42035720&guid=fd9e7cea-1780-4085-afe3-26c518e512f0&scheme=1)

[Supporting Information](https://www2.cloud.editorialmanager.com/pone/download.aspx?id=42035720&guid=fd9e7cea-1780-4085-afe3-26c518e512f0&scheme=1)

[S1_SPSS_Knowledge_Of_ODS_PLOS.sav](https://www2.cloud.editorialmanager.com/pone/download.aspx?id=42035720&guid=fd9e7cea-1780-4085-afe3-26c518e512f0&scheme=1)
