# Supplementary material for: Maternal knowledge of obstetric danger signs and related factors among antenatal care attendees in Hargeisa, Somaliland: A cross-sectional analysis
Source: PLoS One. 2026 Jul 15;21(7):e0353778. doi: 10.1371/journal.pone.0353778 (PMC13372116; doi:10.1371/journal.pone.0353778)
Supplement: Attachment — Submitted filename: Response_to_Reviewers_auresp_2.docx [file pone.0353778.s007.docx]

**Response to Reviewers**

We would like to sincerely thank the Journal staffs, Editor and the Reviewer for their valuable comments and constructive feedback. We have carefully revised the manuscript accordingly and believe that the changes have significantly improved its clarity, rigor, and overall quality. All comments have been addressed point-by-point below.

**Responses to Journal requirements**

1. If the reviewer comments include a recommendation to cite specific previously published works, please review and evaluate these publications to determine whether they are relevant and should be cited. There is no requirement to cite these works unless the editor has indicated otherwise.
   **Response:** We thank you for this guidance. None of the reviewers recommended additional references for citation. Nevertheless, we carefully reviewed the literature and confirmed that all references cited in the manuscript are relevant and appropriate to the study.
2. Please review your reference list to ensure that it is complete and correct. If you have cited papers that have been retracted, please include the rationale for doing so in the manuscript text, or remove these references and replace them with relevant current references. Any changes to the reference list should be mentioned in the rebuttal letter that accompanies your revised manuscript. If you need to cite a retracted article, indicate the article’s retracted status in the References list and also include a citation and full reference for the retraction notice.

**Response:** We carefully reviewed the entire reference list to ensure completeness, accuracy and consistency. We confirmed that none of the cited references have been retracted. We also corrected two reference lists (10 and 11), and verified that all references conform to the journal’s formatting requirements.

**Response to Reviewers:
Responses to Reviewer #3**

The manuscript is well written and easy to follow. The methodology is sound and adequately addresses all relevant areas. The author is kindly advised to ensure that the manuscript has not been submitted to any other journal, in accordance with established publication ethics.

**Response:** We thank the reviewer for this positive assessment of our work. We confirm that this manuscript is not under consideration by any other journal. We fully adhere to the publication ethics and submission policies of PLOS ONE.

**Responses to reviewer #4**

1. Carefully reviewed the reference list
2. Identified the repeated citation.
3. The duplicate reference has been removed, and the reference list has been updated accordingly.
4. All in-text citations have been checked and renumbered to ensure consistency and accuracy throughout the manuscript
5. Spellings and grammatical errors shouls be corrected
6. Attach the ethical clearance for the study

**Responses:** Thank you. We have carefully reviewed and updated the entire reference list once again to ensure accuracy and consistency. We thoroughly proofread the entire manuscript and corrected spelling, grammatical, punctuation, and minor language issues throughout the manuscript to improve clarity and readability. Moreover, we have already attached the ethical approval letter as a supplementary file.

Thank you for your consideration.

**Sincerely**,

Abdeta Muktar Ahmed (On behalf of all authors)
